# Synthesis of tripodal catecholates and their immobilization on zinc oxide nanoparticles

**DOI:** 10.3762/bjoc.11.77

**Published:** 2015-05-07

**Authors:** Franziska Klitsche, Julian Ramcke, Julia Migenda, Andreas Hensel, Tobias Vossmeyer, Horst Weller, Silvia Gross, Wolfgang Maison

**Affiliations:** 1 Department of Chemistry, University of Hamburg, Institute of Pharmaceutical and Medicinal Chemistry, Bundesstr. 45, 20146 Hamburg, Germany; 2 IENI-CNR, Department of Chemical Sciences, University of Padova, INSTM, Via Marzolo 1, 35131 Padova, Italy; 3 Department of Chemistry, University of Hamburg, Institute of Physical Chemistry, Grindelallee 117, 20146 Hamburg, Germany

**Keywords:** bifunctional anchors, catecholates, multivalency, poly(ethylene glycol), ZnO nanoparticles

## Abstract

A common approach to generate tailored materials and nanoparticles (NPs) is the formation of molecular monolayers by chemisorption of bifunctional anchor molecules. This approach depends critically on the choice of a suitable anchor group. Recently, bifunctional catecholates, inspired by mussel-adhesive proteins (MAPs) and bacterial siderophores, have received considerable interest as anchor groups for biomedically relevant metal surfaces and nanoparticles. We report here the synthesis of new tripodal catecholates as multivalent anchor molecules for immobilization on metal surfaces and nanoparticles. The tripodal catecholates have been conjugated to various effector molecules such as PEG, a sulfobetaine and an adamantyl group. The potential of these conjugates has been demonstrated with the immobilization of tripodal catecholates on ZnO NPs. The results confirmed a high loading of tripodal PEG-catecholates on the particles and the formation of stable PEG layers in aqueous solution.

## Introduction

**Figure 1:**
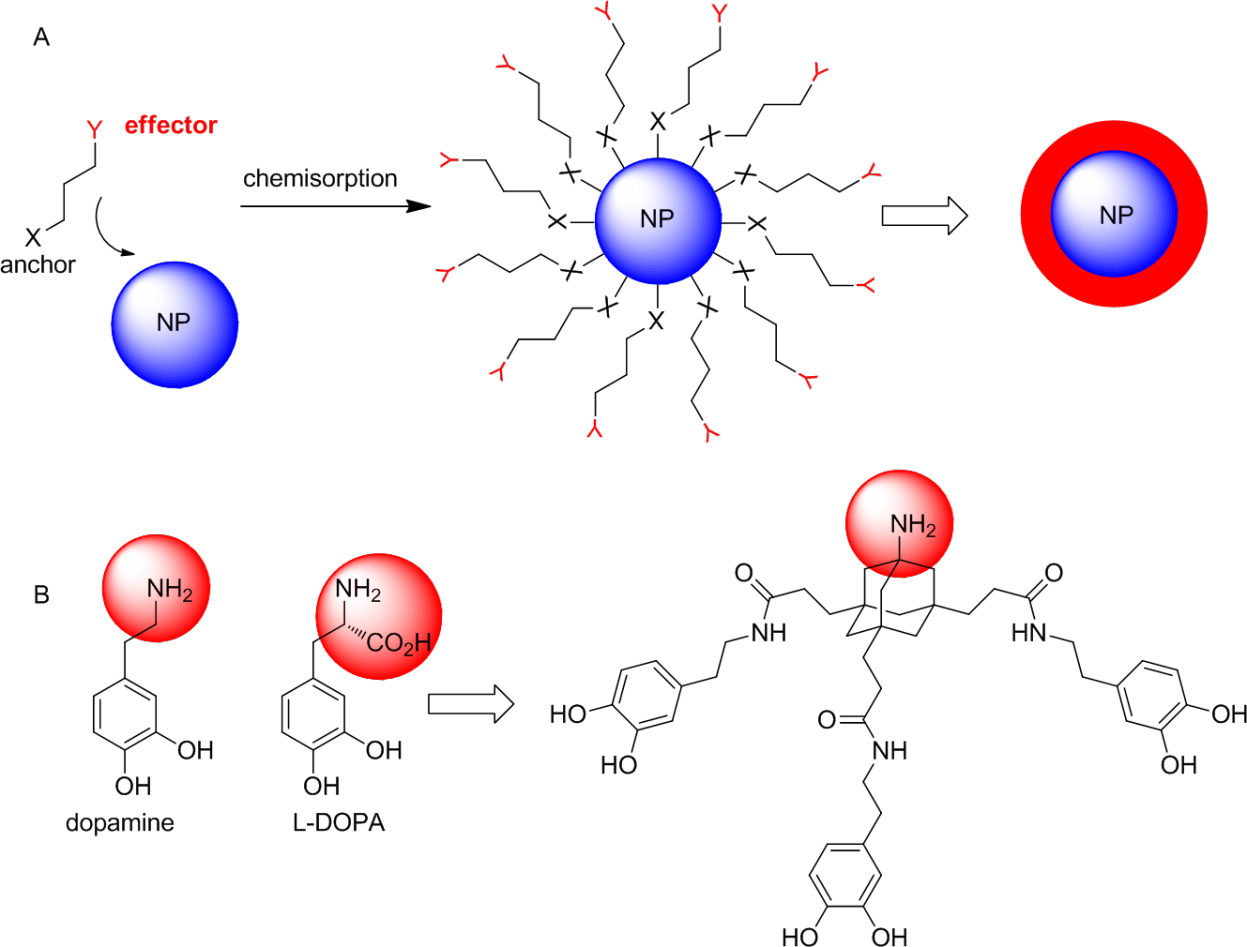
A) Schematic drawing of a bifunctional anchor molecule and its immobilization on a nanoparticle (NP); B) tripodal catechol derivative, derived from the native bifunctional anchors dopamine and L-DOPA.

An elegant approach to generate tailored materials and nanoparticles is the formation of molecular monolayers by chemisorption of bifunctional anchor molecules (Figure 1A) [1]. The effectivity of this approach depends critically on the choice of a suitable anchor molecule. For most applications the anchor needs to be modular and should have functional groups for conjugation of effector molecules via high-yielding and robust chemical transformations. On the other hand, the anchor moiety needs to form a stable (in most cases covalent) connection to the target surface. Various bifunctional anchors have been reported for immobilization on different materials and nanoparticles. Basically, silane derivatives are used for glass surfaces [2–3], thiols for noble metal surfaces [4], carboxylates [5] and phosphates [6] as well as phosphonates [7] for metal and metal oxide surfaces. In addition, bifunctional catechols like dopamine or DOPA (L-3,4-dihydroxyphenylalanine, Figure 1B), have received considerable interest as anchor groups for important metal surfaces such as titanium oxide, iron oxide and stainless steel [8–11]. Immobilization of catecholates was inspired by mussel-adhesive proteins (MAPs) and bacterial siderophores [12].

However, many applications of catecholate immobilization in physiological media are compromised by continuous leaching of grafted material which is a consequence of reversible binding at neutral and slightly acidic pH. Multivalent catecholates, such as MAPs or oligo-DOPA, overcome this drawback of simple catecholate derivatives and show increased binding affinities to metal surfaces. They are therefore attractive anchors for durable immobilizations on metal surfaces in aqueous media [13]. We have recently reported non-peptidic trimeric catecholates and have demonstrated their potential to form stable molecular monolayers on bulk TiO_2_ and stainless steel surfaces in aqueous environment [14–15]. In the present work, we describe the synthesis of effector-conjugates of tripodal catecholates and their immobilization on ZnO NPs.

## Results and Discussion

Zinc oxide belongs to the most intensively investigated inorganic compounds, due to its outstanding functional properties combined with manifold morphologies, no toxicity and easy preparation [16]. It is a piezoelectronic semiconductor with a high exciton binding energy (60 meV) and a wide band-gap (3.37 eV) at room temperature [17–18]. ZnO is therefore employed in (bio-)sensors [19], ultraviolet (UV) light-emitting diodes [20], UV laser diodes [21] and in the field of catalysis [22–23]. ZnO exists in several morphologies such as nanowires, nanotubes, nanoparticles, nanoplatelets and nanowhiskers [24]. Colloidal ZnO nanoparticles are especially interesting because of their functional properties. Classical methods of colloid chemistry can be used for the preparation of colloidal suspensions [25] and various paths to obtain ZnO colloids have been reviewed by Spanhel [26]. Suitable methods for the synthesis of pure and doped ZnO NPs involve colloidal, sol–gel or solvo-/hydrothermal methods [27], microemulsion and miniemulsion methods [28] or non-aqueous sol–gel routes [29]. Recently, some of us established an easy and fast procedure to obtain nanocrystalline ZnO nanoparticles, which was applied to prepare the ZnO nanoparticles used in this work [30].

Immobilization of effector molecules on ZnO NPs has been accomplished with oxygen donors such as carboxylic acids. Bifunctional derivatives bearing an additional effector moiety may be used to generate stable particles with tailored properties, good solubility and biocompatibility. Suitable effectors in this context are PEG [31–33], zwitterions [34–35] or polyglycerols [36–37] which, when immobilized on NPs, may be used to tune their pharmacokinetic properties [38–39]. The resulting particles show a reduced tendency towards plasma protein and tissue binding, both important factors influencing elimination and tissue distribution of biological imaging reagents. Based on our good experiences with the immobilization of tripodal catecholates on TiO_2_ and steel, we explored their use for the functionalization of ZnO NPs.

### Synthesis of tripodal catecholates

**Scheme 1:**
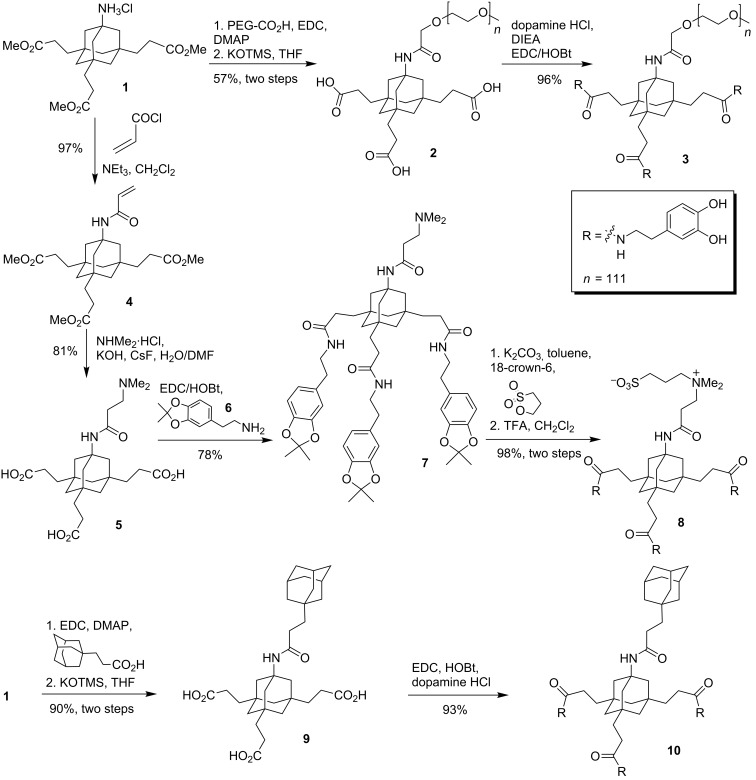
Synthesis of tripodal catecholates for surface immobilization. PEG-triscatecholate **3** was synthesized from **1** according to literature [31]. Abbreviations: PEG = poly(ethylene glycol) (5 kDa); EDC = 1-ethyl-3-(3-dimethylaminopropyl)carbodiimide; DMAP = 4-(dimethylamino)pyridine; HOBt = hydroxybenzotriazole; TFA = trifluoroacetic acid, KOTMS = potassium trimethylsilanolate, DIEA = *N,N*-diisopropylethylamine.

A common synthetic precursor for the synthesis of suitable tripodal catecholates is the AB_3_-scaffold **1** [40–42] (Scheme 1) which is readily available in a few steps from adamantane as a cheap starting material [43]. Amine **1** was coupled to a commercially available PEG-carboxylate (5 kDa) with EDC/DMAP. The resulting PEG-conjugate was treated with KOTMS to remove the methyl esters to give tricarboxylic acid **2** in good 57% yield for the two-step procedure. In a last step, dopamine was coupled to the free carboxylic acids to give PEG-triscatecholate **3** in excellent yield [31]. This PEG-conjugate is ready for the immobilization on NPs and may be used to generate biopassive (stealth) particles for biomedical applications.

As an alternative to PEG as an effector moiety, we tried to conjugate the triscatecholates to a sulfobetaine group. Like PEG, these zwitterionic moieties have been used frequently to confer biopassive properties to metal surfaces but are less prone to oxidative degradation [35]. The synthesis started again from AB_3_-scaffold **1** which was acylated with acryloyl chloride to give acrylamide **4**. Treatment of **4** with dimethylamine and excess KOH leads to the nucleophilic addition of the amine and saponification of the methyl esters in one step to give the free acid **5** after acidic work-up. Subsequent coupling of **5** to dopamine acetonide **6** with EDC and HOBt gave the protected triscatecholate **7** in good yield. The sulfobetaine was then generated by treatment of **7** with 1,3-propane sultone and the acetonides were cleaved with TFA to give the free triscatecholate **8**. Following the same synthetic strategy, the hydrophobic derivative **10** bearing an additional adamantyl group as an effector was prepared. This triscatecholate might be useful for the construction of diamandoid hydrophobic coatings [44] or for the reversible attachment of cyclodextrins to NPs by the formation of cyclodextrin/adamantane inclusion complexes [45].

**Scheme 2:**
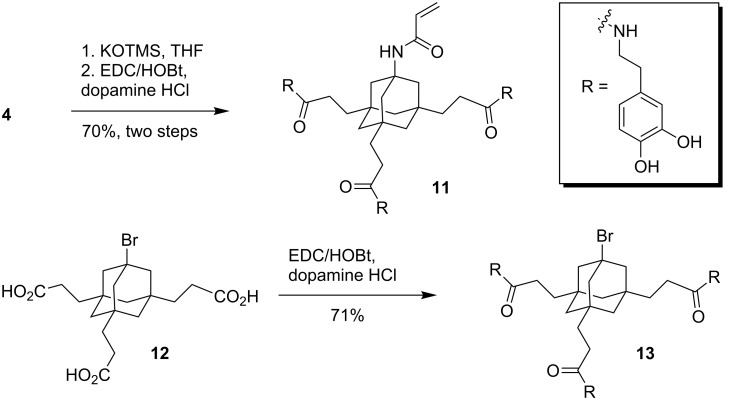
Synthesis of tripodal catecholate platforms **11** and **13** for surface functionalization.

Alternatively, acrylamide **4** and bromide **12** [42] were converted to the corresponding triscatecholates **11** and **13** by coupling to dopamine (Scheme 2). The resulting triscatecholates **11** and **13** may be used as synthetically flexible platforms for functionalizations of surfaces via either nucleophilic addition (to the Michael acceptor in **11**) or radical chemistry after immobilization.

### Immobilization on ZnO NPs

**Figure 2:**
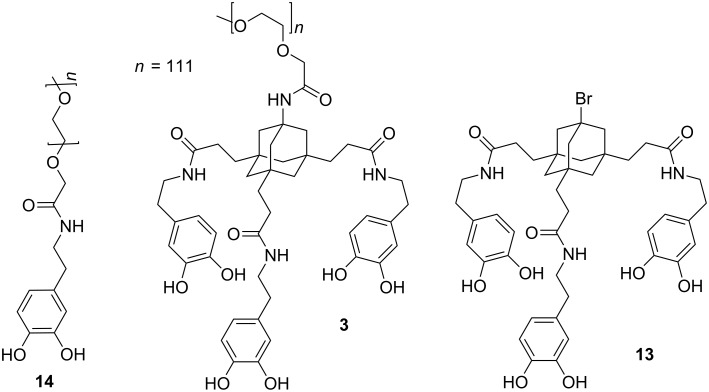
Catecholates for the immobilization on ZnO NPs.

Three different catecholates were selected to study the binding properties to ZnO NPs (Figure 2). Monomeric PEG-catecholate **14** [46] and the tripodal homologue **3** were chosen to study the stability of the coatings and the particles in aqueous solution depending on the valency of the catecholate. Bromotriscatecholate **13** was chosen as a hydrophobic analogue to **3**.

ZnO particles were synthesized according to a literature known procedure from Zn(acac)_2_ [30].

**Figure 3:**
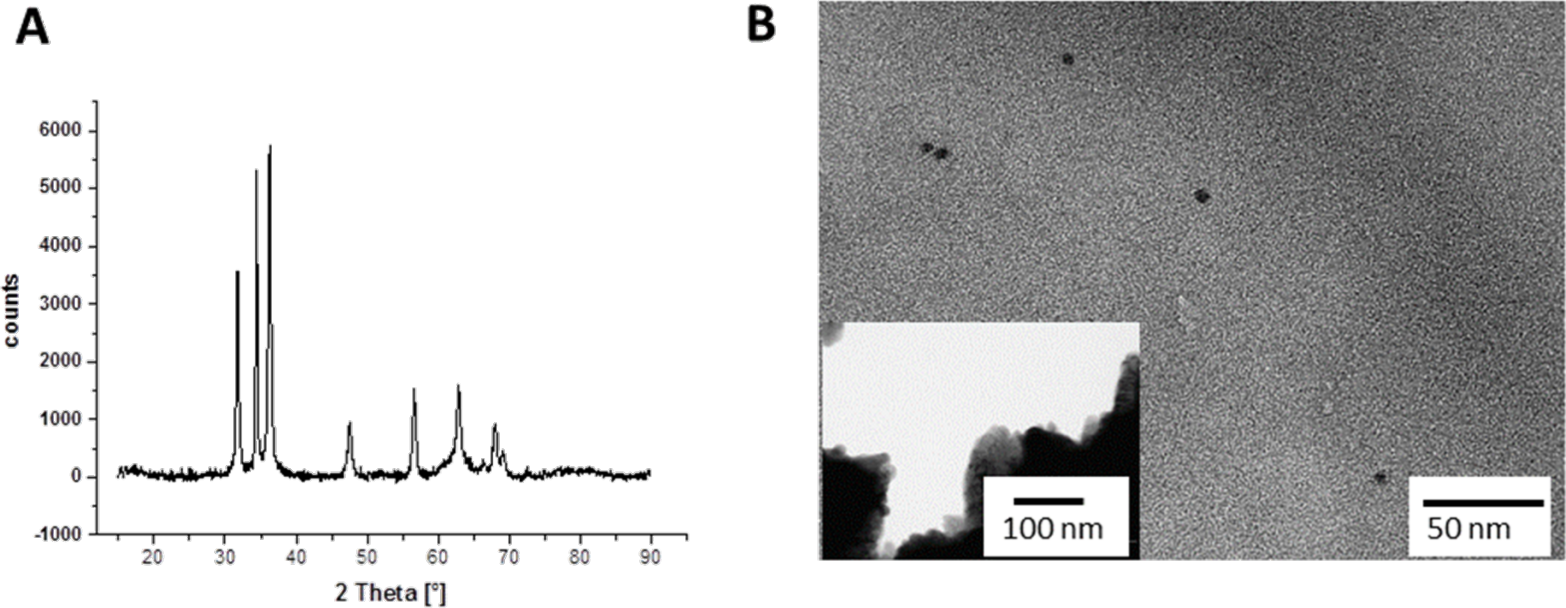
A) XRD pattern of ZnO NPs obtained by the colloidal suspension of Zn(acac)_2_. B) TEM image of pure ZnO nanoparticles.

Powders separated by the centrifugation of the precursor suspensions were investigated by X-ray diffraction to confirm the formation of crystalline materials. The XRD pattern confirms the selective formation of pure ZnO wurtzite already at room temperature without the need of any further thermal treatment (Figure 3A). This data is in agreement with TEM micrographs, indicating the presence of spherical particles with an average diameter of 6 nm next to larger crystal aggregates (Figure 3B).

The particles were coated using solutions of monomeric PEG-catecholate **14** and the tripodal catecholates **3** and **13** in a concentrated 3-morpholinopropanesulfonic acid (MOPS) buffer at pH 10 [14,31]. Under these conditions, the catechol moieties were reasonably stable and only small amounts (5%) of the corresponding oxidized quinones were detectable by NMR in the solutions after 24 h. The ZnO NPs were treated with the buffered catecholate solutions for 12 h, isolated by centrifugation, washed with a small amount of water (pH 7) and MeOH and freeze-dried before analysis by XRD, IR, HRTEM-EDX and TGA. A reference probe of ZnO NPs was treated the same way, but no catecholate was added to the buffer.

**Figure 4:**
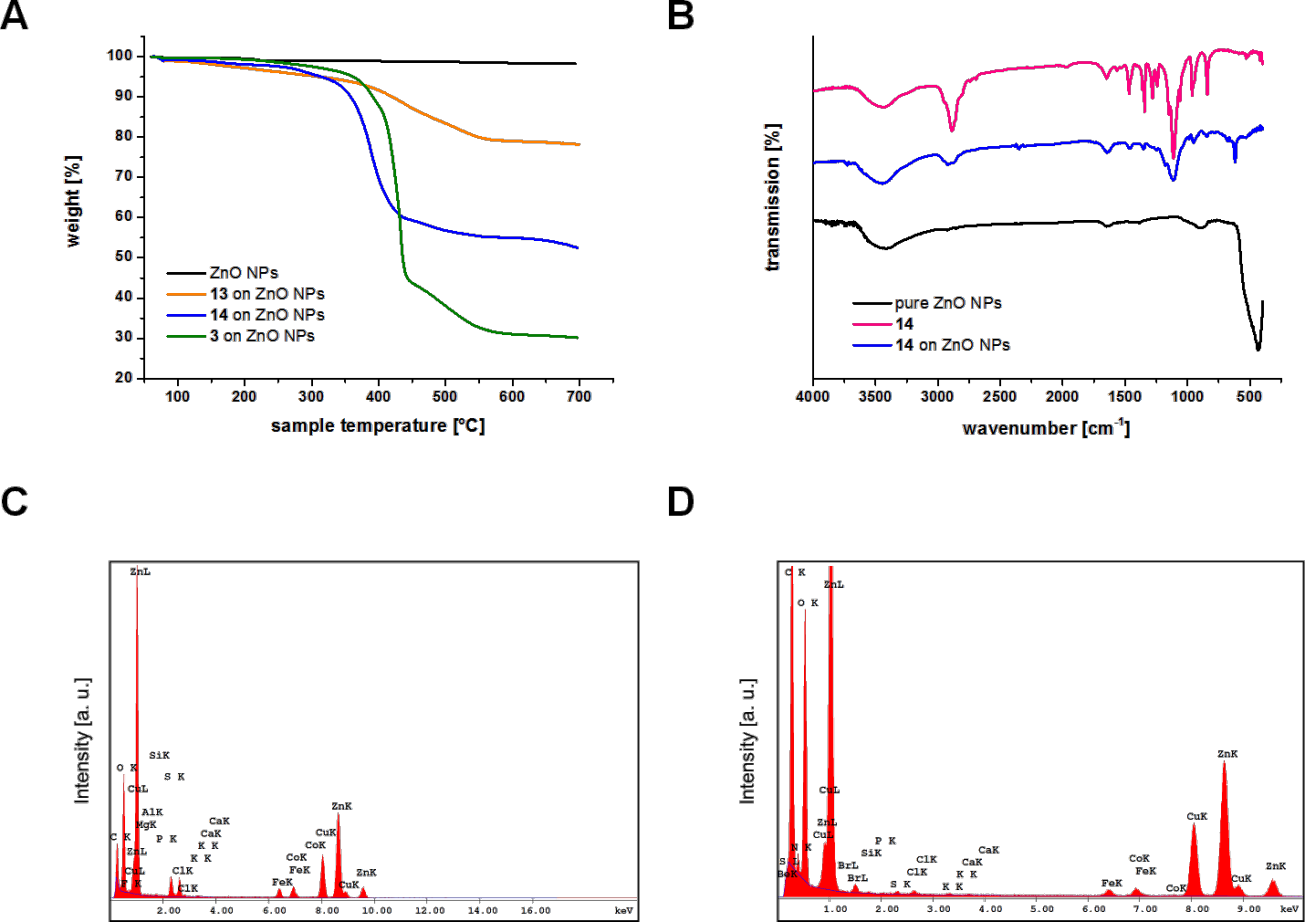
A) TGA data of catecholates **3**, **13** and **14** immobilized on ZnO NPs: pure ZnO NPs treated with MOPS buffer (black line), bromo-triscatecholate **13** on ZnO NPs (after washing with water and MeOH, orange line), monomeric PEG-catecholate **14** on ZnO (after centrifugation, blue line) and tripodal PEG-catecholate **3** on ZnO (after washing with water and MeOH, green line). B) FTIR spectra of pure ZnO NPs (before immobilization, black line), monomeric PEG-catecholate **14** (pink line) and monomeric PEG-catecholate **14** immobilized on ZnO NPs (blue line). C) EDX spectrum of pure ZnO NPs. D) EDX spectrum of bromo-triscatecholate **13** immobilized on ZnO.

3-Morpholinopropanesulfonate, the ingredient of the MOPS buffer, showed only a weak affinity for ZnO NPs according to the corresponding TGA curve in Figure 4A and EDX (Figure 4C). Sulfonates have been described as ZnO binders before [47–48]. However, the binding affinity of 3-morpholinopropanesulfonate to ZnO NPs is low and most of the ligand is eliminated by washing following the immobilization.

In contrast, TGA indicated a high loading of the particles with both the monomeric PEG-catecholate **14** (48 wt % loading) and the tripodal catecholates **3** (70 wt % loading) and **13** (17 wt % loading). The latter two values are close to the theoretical maximum loading of 63 wt % (for **3**) and 20 wt % (for **13**, note the dramatically lower mass of **13** compared to PEG-conjugates **3** and **14**), which was calculated for an ideal particle of 6 nm diameter and 0.25 nm^2^ coverage per catecholate residue [49]. The loading of monomeric PEG-catecholate **14** on ZnO NPs is significantly lower compared to the calculated maximum loading of 86 wt %. This indicates that a large fraction of **14** is already lost during the first washing procedure, reflecting the reversible binding of monomeric catecholates to metal oxides, as mentioned above. Successful immobilization was also confirmed by IR as showcased for monomeric PEG-catecholate **14** in Figure 4B (for IR spectra of immobilized trimeric catecholates **3** and **13** see Supporting Information File 1).

**Figure 5:**
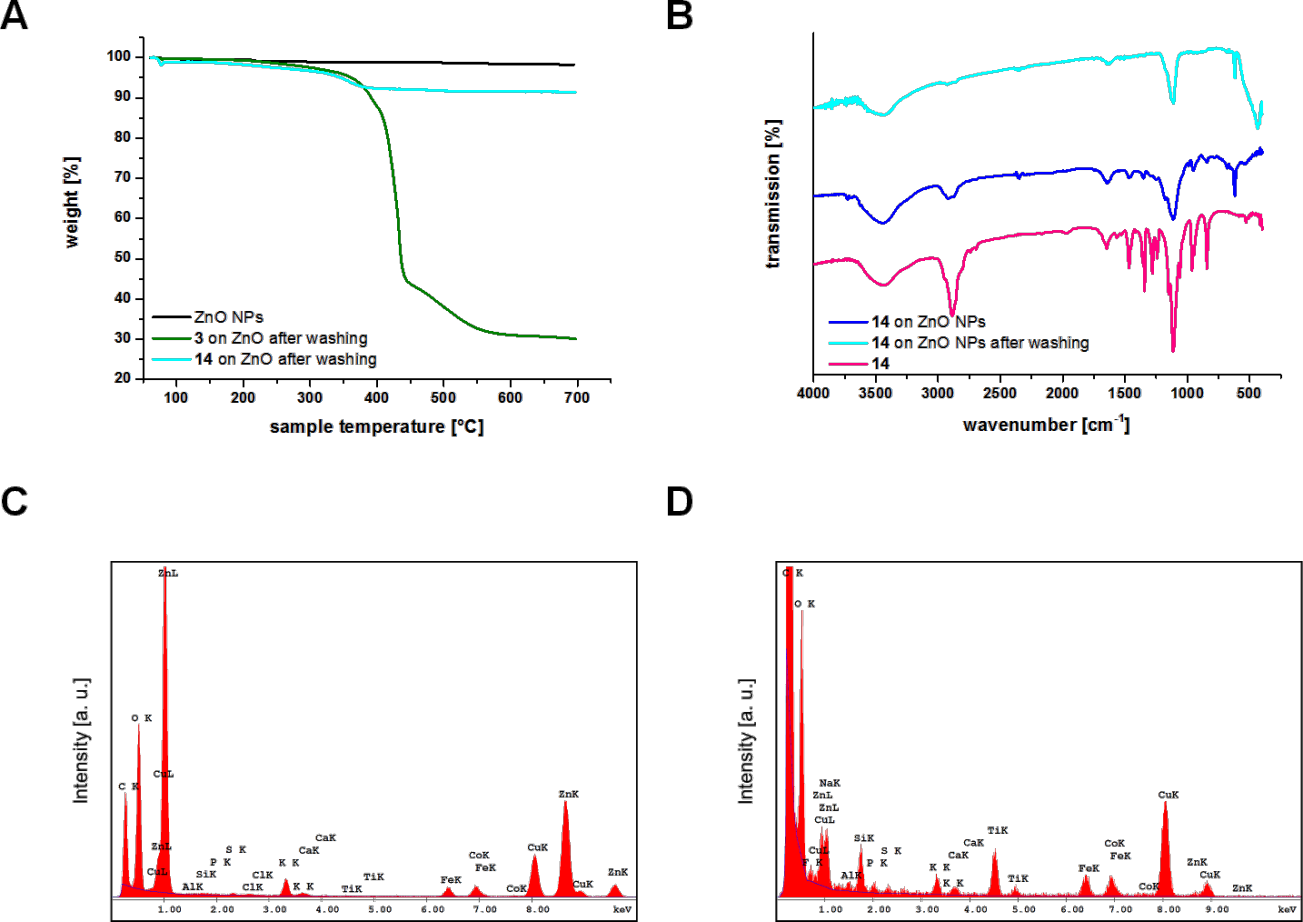
A) TGA data of catecholates **3** and **14** immobilized on ZnO NPs: pure ZnO NPs treated with MOPS buffer (black line), monomeric PEG-catecholate **14** on ZnO after washing with water and MeOH for three times (cyan line) and tripodal PEG-catecholate **3** on ZnO after washing with water and MeOH for three times (green line). B) FTIR spectra of monomeric PEG-catecholate **14** immobilized on ZnO after washing twice with water and MeOH (cyan line), monomeric PEG-catecholate **14** immobilized on ZnO after centrifugation from MOPS buffer (blue line) and monomeric PEG-catecholate **14** (pink line). C) EDX spectrum of monomeric PEG-catecholate immobilized on ZnO NPs after washing with water and MeOH. D) EDX spectrum of tripodal PEG-catecholate immobilized on ZnO NPs after washing with water and MeOH.

This effect is increasingly important if the coated particles are handled in aqueous solution. After three successive rounds of washing with water and MeOH, almost all of the monomeric PEG-catecholate **14** is lost from the particles as determined by TGA (Figure 5A) and confirmed qualitatively by comparison of the different intensity of the carbon peaks in the EDX spectra of monomer **14** and trimer **3** on ZnO (Figure 5C and D). In contrast, loading of the tripodal PEG-catecholate **3** is retained at about 70 wt %. The comparably lower loss of catecholate loading confirms the ability of our triscatecholates to form stable layers on ZnO NPs and parallels our previous observations on TiO_2_ and stainless steel surfaces [31].

**Figure 6:**
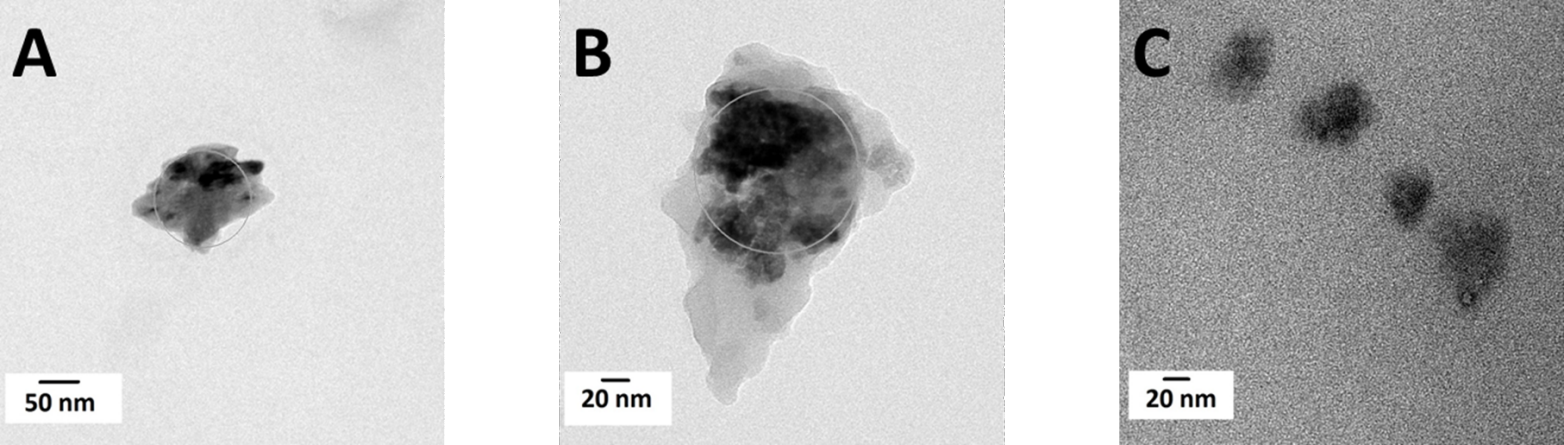
TEM images of ZnO NPs. A) ZnO NPs coated with monomeric PEG-catecholate **14** after washing with water and MeOH for three times. B) ZnO NPs coated with bromo-triscatecholate **13** after washing with water and MeOH for three times. C) ZnO NPs coated with tripodal PEG-catecholate **3** after washing with water and MeOH for three times.

The observed difference in catechol loading has an impact on the stability of the ZnO NPs in water. The TEM images in Figure 6 show the coated particles after three rounds of washing with water and MeOH. Homogenous isolated spherical particles of about 25 nm diameter are observed for tripodal PEG-catecholate **3** (Figure 6C). This compares well to the expected size of 6 nm NPs coated with a 5 kDa PEG [50]. In contrast, the particles initially coated with monomeric PEG-catecholate **14** form larger aggregates (Figure 6A). As expected, particles coated with the hydrophobic tripodal catecholate **13** show the same tendency for aggregation in aqueous solution (Figure 6B).

## Conclusion

We report here the synthesis of new tripodal catecholates as valuable multivalent anchor molecules for immobilization on metal surfaces and nanoparticles. These catecholate anchors make use of a biomimetic covalent immobilization concept as found for example in mussel adhesion proteins. Our tripodal catecholate anchors are bifunctional and have been conjugated to various effector molecules such as PEG, a sulfobetaine and an adamantyl group, thus evidencing the feasibility and versatility of the developed approach. The resulting effector-catecholate conjugates are useful for the generation of biopassive (stealth) surfaces (PEG and sulfobetaine) or switchable hydrophobic/hydrophilic layers (reversible formation of adamantane/cyclodextrin inclusion complexes) on bulk metal surfaces or NPs.

The potential of these conjugates has been demonstrated through the immobilization of the tripodal PEG-catecholate **3** on ZnO NPs and a comparison with the monovalent PEG-catecholate **14**. The results confirmed a high loading of tripodal PEG-catecholate **3** on the particles and the formation of stable catecholate layers in aqueous solution. Immobilization of the monomeric PEG-catecholate **14** was also successful. However, the monomeric catecholate **14** is rapidly eliminated by treatment of the coated particles with water, thus highlighting a much lower stability.

In summary, effector conjugates of tripodal catecholates such as **3** and **13** form stable layers on ZnO NPs even in water. The results reported here confirm our previous studies of tripodal catecholates and their immobilization on TiO_2_ and stainless steel.

## Experimental

### Synthesis

The following compounds were synthesized according to literature procedures: **1** [42], **2** [31], **3** [31], **12** [42] **14** [46].

### Thermogravimetric analysis

The TGA data were obtained with a Pyris 1 TGA of Perkin Elmer under Nitrogen gas flow. The samples were heated at 80 °C isothermally for 10 minutes and subsequently heated from 80 °C to 700 °C at a rate of 10 °C per minute. The experiments were carried out at least two times.

### Fourier transformation infrared spectroscopy

IR spectra were measured on a Jasco FTIR 4100 device as a disc of anhydrous potassium bromide purchased from Merck.

### TEM analysis

For TEM analysis, the functionalized particles were dispersed in MeOH and dropped onto 400-mesh carbon-coated TEM copper grids. The samples were analyzed using a JEOL JEM-1011 microscope, equipped with a LaB6 cathode and operated at 100 kV.

### HRTEM and EDX analysis

For high resolution TEM (HRTEM) and energy-dispersive X-ray analysis (EDX), the functionalized particles were dispersed in MeOH and transferred to carbon-coated TEM grids. The samples were analyzed using a Philips CM 300 microscope, operated at 300 kV.

### XRD analysis

For XRD analysis, the functionalized particles were dispersed in MeOH, dropped on a standard crystal Si support. Then the solvent was evaporated. The samples were analyzed using a Philips X`Pert PRO MPD diffractometer (Cu Kα radiation, variable entrance slit, Bragg–Brentano geometry, secondary monochromator).

## Supporting Information

File 1 Experimental procedures, additional analytical data and NMR spectra.
