# Supplementary material for: Synthesis of tripodal catecholates and their immobilization on zinc oxide nanoparticles
Source: Beilstein J Org Chem. 2015 May 7;11:678–86. doi: 10.3762/bjoc.11.77 (PMC4464264; doi:10.3762/bjoc.11.77)
Supplement: File 1 — Experimental procedures, additional analytical data and NMR spectra. [file Beilstein_J_Org_Chem-11-678-s001.pdf]

Supporting Information

for

Synthesis of tripodal catecholates and their immobilization on zinc oxide nanoparticles

Franziska Klitsche[1], Julian Ramcke[1], Julia Migenda[2], Andreas Hensel[3], Tobias Vossmeyer[3], Horst Weller[3], Silvia Gross*[2] and Wolfgang Maison*[1]

Address: [1]Department of Chemistry, University of Hamburg, Institute of Pharmaceutical and Medicinal Chemistry, Bundesstr. 45, 20146 Hamburg, Germany, [2]IENI-CNR, Department of Chemical Sciences, University of Padova, INSTM, Via Marzolo 1, 35131 Padova, Italy and [3]Department of Chemistry, University of Hamburg, Institute of Physical Chemistry, Grindelallee 117, 20146 Hamburg, Germany

Email: Silvia Gross - silvia.gross@unipd.it; Wolfgang Maison - maison@chemie.uni-hamburg.de

*Corresponding author

Experimental procedures, additional analytical data and NMR spectra

Table of content

Experimental procedures and analytical data for compounds **4**, **5, 7–11, 13**…S2–S10

NMR spectra of compounds **4**, **5, 7–11, 13**……………………………………..S11–S21

Supplementary data and general notices of the immobilization experiments..S22–S36

References..S37

Experimental procedures and analytical data for compounds 4, 5, 7–11, 13

General methods: Infrared spectra (IR) of the substances were measured with a Bruker Tensor 37 ATR-FTIR-spectrophotometer. Spectra were recorded from 400 to 4000 cm^{-1} at room temperature. Mass spectra were obtained with a Micro-TOF-Q instrument equipped with an ESI-source. Measurements were conducted in positive or negative mode. NMR spectra were recorded on a Bruker FOURIER 300 at 300 MHz (^{1}H), and 75 MHz (^{13}C). The spectra were referenced to residual non-deuterated solvent signal. The chemical shifts δ are noted in ppm and the multiplicity of the signals is designated as s (singlet), d (doublet), dd (doublet of doublet), t (triplet), q (quartet), sext (sextet) and m (multiplet). Signal identification was carried out with adequate 1D and 2D-NMR experiments (e.g., HSQC, HMBC and COSY). The applied solvents were distilled prior to use and all reagents were reagent grade and used without further purification unless otherwise specified. Column chromatography was carried out in glass columns with the use of silica (60–200 µm) purchased by Macherey-Nagel GmbH & Co KG with the appropriate mentioned solvent. The determination of the R_f-values was performed with thin-layer chromatography on aluminium sheets (ALUGRAM Xtra SIL G/UV 254) purchased by Macherey-Nagel GmbH & Co KG. The detection of the UV active substances was conducted at a wavelength of 254 nm whereas UV inactive substances were detected with cerium sulfate solution. The poly(ethylene glycol) derivatives were stained with ethanolic sulfuric acid prior to analysis. Melting points were measured with the Büchi M-560 device. The elementary analyses have been performed at an EA 1108 CHNS-O of the manufacturer Carlo Erba through the service devision of the Department of Chemistry at the University of Hamburg.

Acrylamide 4: Triester **1** [1] (1.15 g; 2.60 mmol) was dissolved in CH_2Cl_2 (40 mL) and NEt_3 (4.00 mL; 28.6 mmol) were added at 0 °C. The solution was treated with acryloyl chloride (0.53 mL; 6.5 mmol) in CH_2Cl_2 (20 mL) over 30 minutes and stirred for 17 h at rt. The solution was washed with 2 M aqueous HCl (50 mL) dried over Na_2SO_4, filtered and the solvent was evaporated. The title compound was obtained as an orange oil (1.17 g; 2.52 mmol; 97%). **^{1}H-NMR** (300 MHz, $CDCl_3$): δ = 6.17 (dd, 1H, $^2J_{H,H}$ = 1.9 Hz, $^3J_{H,H}$ = 17.1 Hz, 11[E to 10]-H); 5.98 (dd, 1H, $^3J_{H,H}$ = 10.2 Hz, $^3J_{H,H}$ = 17.1 Hz, 10-H); 5.53 (dd, 1H, $^2J_{H,H}$ = 1.9 Hz, $^3J_{H,H}$ = 10.2 Hz, 11[Z to 10]-H); 3.61 (s, 9H, 8-H); 2.22 (t, 6H, $^3J_{H,H}$ = 8.1 Hz, 6-H); 1.63 (s, 6H, 2-H); 1.49 (t, 6H, $^3J_{H,H}$ = 8.1 Hz, 5-H); 1.13 (d, 3H, $^2J_{H,H}$ = 12.0 Hz 4a-H); 1.02 (d, 3H, $^2J_{H,H}$ = 12.0 Hz 4b-H). **^{13}C-NMR** (75 MHz, $CDCl_3$): δ = 174.5 (C7); 164.8 (C9); 131.7 (C11); 126.0 (C10); 53.9 (C8); 51.7 (C1); 45.1 (C4); 44.6 (C2); 37.4 (C5); 34.8 (C3); 28.1 (C6). HRMS-ESI: *m/z* $[M+Na]^+$= calc. $C_{25}H_{37}NO_7Na^+$, 486.2468; found 486.2468. R_f: 0.80 (CH_2Cl_2/MeOH; 9:1; v/v).

Triacid 5: Acrylamide **4** (250 mg; 0.539 mmol) were dissolved in water (50 mL). Dimethylammonium chloride (88.0 mg; 1.08 mmol), KOH (305 mg; 5.40 mmol) and CsF (8.0 mg; 54 µmol) were added and stirred for 17 h at rt. The solution was treated with 1 M aqueous HCl to adjust a pH-value of 1. After freeze-drying the crude product was dissolved in THF (50 mL), stirred for 5 minutes at 40 °C, filtered and the solvent was evaporated. The title compound was obtained as a yellow solid (204 mg; 437 µmol; 81%). **^{1}H-NMR** (300 MHz, Methanol-d_4): δ = 3.35 (t, 2H, $^3J_{H,H}$ = 6.7 Hz, 9-H); 2.89 (s, 6H, 11-H); 2.66 (t, 2H, $^3J_{H,H}$ = 6.7 Hz, 10-H); 2.30 (t, 6H, $^3J_{H,H}$ = 8.1 Hz, 6-H); 1.64 (s, 6H, 2-H); 1.52 (t, 6H, $^3J_{H,H}$ = 8.1 Hz, 5-H); 1.18 - 1.07 (m, 6H, 4-H). **^{13}C-NMR** (75 MHz, Methanol-d_4): δ = 176.3 (C7); 171.1 (C8); 55.4 (C1); 55.1 (C10); 46.0 (C4); 45.4 (C2); 43.5 (C11); 38.8 (C5); 35.9 (C3); 30.9 (C9); 28.9 (C6). HRMS-ESI: *m/z*

$[M+H]^+$= calc. $C_{24}H_{38}N_2O_7H^+$, 467.2752; found 467.2753. R_f: 0.10 (EtOAc/EtOH; 9:1; v/v).

Protected triscatecholate 7: Triacid **5** (160 mg; 343 µmol) was dissolved in 20 mL DMF and DIEA (*N*,*N*-diisopropylethylamine, 0.10 mL, 548 µmol) were added at 0 °C. The solution was treated with EDC·HCl (217 mg; 1.13 mmol) and HOBt (153 mg; 1.13 mmol) in DMF (5 mL each). The resulting solution was stirred for 30 min at 0 °C and 2-(2,2-dimethylbenzo[*d*][1,3]dioxol-5-yl)ethanamine hydrochloride (**6**) [2] (260 mg; 1.13 mmol) in DMF (5 mL) were added. The mixture was stirred for 72 h at rt and the solvent was removed in vacuo. The resulting residue was dissolved in 2 M aqueous HCl (50 mL) and extracted three times with EtOAc (50 mL each). The combined organics were washed three times with sat. aqueous $KHSO_4$ (15 mL each), dried over Na_2SO_4, filtered and the solvent was evaporated. The remaining solid was purified by flash chromatography on silica (EtOAc/EtOH/NEt_3, 90:10:2 v/v/v) to give the title compound (266 mg; 268 µmol; 78%) as a yellow solid. **^{1}H-NMR** (300 MHz, $CDCl_3$): δ= 7.77 (s, 1H, NH); 6.66 - 6.56 (m, 9H, 15-H, 18-H, 19-H); 5.90 (m, 3H, NH); 3.45 - 3.38 (m, 6H, 12-H); 2.77 - 2.66 (m, 8H, 9-H, 13-H); 2.41 - 2.39 (m, 8H, 10-H, 11-H); 2.07 (t, 6H, $^3J_{H,H}$ = 7.1 Hz, 6-H); 1.65 (s, 18H, 21-H); 1.57 (s, 6H, 2-H); 1.51 - 1.45 (m, 6H, 5-H); 1.16 - 0.99 (m, 6H, 4-H). **^{13}C-NMR** (75 MHz, $CDCl_3$): δ = 173.5 (C7); 170.8 (C8); 147.7 (C16); 146.1 (C17); 132.0 (C14); 121.0 (C15); 117.9 (C20); 108.9 (C18); 108.2 (C19); 53.5 (C1); 45.9 (C4); 45.5 (C9); 44.8 (C10); 44.2 (C2); 41.0 (C12); 38.5 (C5); 35.5 (C13); 34.9 (C3); 30.5 (C6); 25.9 (C21). HRMS-ESI: *m/z* $[M+H]^+$= calc. $C_{57}H_{77}N_5O_{10}H^+$, 992.5743; found 992.5743. HRMS-ESI: *m/z* $[M+Na]^+$= calc. $C_{57}H_{77}N_5O_{10}Na^+$, 1014.5563; found 1014.5555. Melting point: 126 °C. R_f: 0.20 (CH_2Cl_2/MeOH, 9:1 v/v).

Zwitterionic triscatecholate 8: Protected triscatecholate **7** (100 mg; 102 µmol) was dissolved in toluene (50 mL), K_2CO_3 (6.9 mg; 50 µmol), 1,3-propanesultone (24 mg;

0.20 mmol) and 18-crown-6 (26 mg; 0.10 mmol) were added and stirred for 72 h at 120 °C. The suspension was filtered, the solid dried and dissolved in THF. The suspension was filtered and the solvent was evaporated. The intermediate protected zwitterionic triscatecholate was obtained as a yellow solid (111 mg; 99.6 µmol; 98%). **^{1}H-NMR** (300 MHz, THF-d_8): δ = 6.62 - 6.57 (m, 9H, 18-H, 21-H, 22-H); 3.55 - 3.53 (m, 2H, 9-H); 3.24 (t, 6H, $^{3}J_{H,H}$ = 7.4 Hz, 15-H); 3.14 (s, 6H, 11-H); 2.86 (t, 2H, $^{3}J_{H,H}$ = 6.9 Hz, 12-H); 2.74 - 2.69 (m, 2H, 10-H); 2.62 (t, 6H, $^{3}J_{H,H}$ = 7.4 Hz, 16-H); 2.54 (t, 2H, $^{3}J_{H,H}$ = 6.7 Hz, 14-H); 2.24 - 2.18 (m, 2H, 13-H); 2.14 - 2.08 (m, 6H, 6-H); 1.64 (s, 6H, 2-H); 1.57 (s, 18H, 24-H); 1.48 - 1.42 (m, 6H, 5-H); 1.19 (d, 3H, $^{2}J_{H,H}$ = 11.8 Hz 4a-H); 1.03 (d, 3H, $^{2}J_{H,H}$ = 11.8 Hz 4b-H). **^{13}C-NMR** (75 MHz, THF-d_8): δ = 175.9 (C7); 170.1 (C8); 148.4 (C19); 146.7 (C20); 133.9 (C17); 122.1 (C18); 118.4 (C23); 109.9 (C21); 108.5 (C22); 71.4 (C10); 71.3 (C12); 54.8 (C1); 51.8 (C11); 48.5 (C9); 46.3 (C4); 44.9 (C2); 42.1 (C15); 39.9 (C5); 36.3 (C16); 35.8 (C3); 31.0 (C6); 30.0 (C24); 24.7 (C14); 19.6 (C13). HRMS-ESI: *m/z* $[M+H]^{+}$= calc. $C_{60}H_{83}N_5O_{13}SH^{+}$, 1114.5768; found 1114.5760. HRMS-ESI: *m/z* $[M+2H]^{2+}$= calc. $C_{60}H_{83}N_5O_{13}SH_2^{2+}$, 557.7932; found 557.7931. Melting point: 281 °C (dec.). R_f: 0.30 (CH_2Cl_2/MeOH, 9:1 v/v).

This intermediate (20 mg; 18 µmol) was suspended in CH_2Cl_2 (10 mL), TFA (1.0 mL) was added and stirred for 24 h at rt. The solvent was evaporated under reduced pressure. The title compound **8** was obtained as a yellow solid (18 mg; 18 µmol; 100%). **^{1}H-NMR** (300 MHz, DMSO-d_6): δ = 8.73 (s, 6H, OH); 7.87 - 7.83 (m, 3H, NH); 7.72 - 7.68 (m, 1H, NH); 6.63 (d, 3H, $^{3}J_{H,H}$ = 7.9 Hz, 21-H); 6.42 (d, 3H, $^{4}J_{H,H}$ = 1.8 Hz, 18-H); 6.40 (dd, 3H, $^{3}J_{H,H}$ = 7.9 Hz, $^{4}J_{H,H}$ = 1.8 Hz, 22-H); 3.50 (t, 2H, $^{3}J_{H,H}$ = 6.5 Hz, 9-H); 3.16 - 3.10 (m, 6H, 15-H); 3.00 - 2.95 (m, 6H, 11-H); 2.50 - 2.46 (m, 6H, 16-H); 2.04 - 1.90 (m, 8H, 6-H, 14-H); 1.58 - 1.47 (m, 6H, 10-H, 12-H, 13-H); 1.37 - 1.23 (m,

12H, 2-H, 5-H); 1.08 - 0.97 (m, 6H, 4-H). **^{13}C-NMR** (75 MHz, DMSO-d_6): δ = 172.4 (C7); 168.8 (C8); 145.0 (C19); 143.4 (C20); 130.2 (C17); 119.1 (C18); 115.9 (C21); 115.4 (C22); 69.8 (C10); 67.0 (C12); 53.2 (C1); 50.7 (C11); 49.8 (C9); 46.7 (C4); 45.0 (C2); 40.5 (C15); 34.6 (C5); 34.4 (C16); 29.8 (C3); 29.5 (C6); 22.4 (C14); 13.9 (C13). HRMS-ESI: *m/z* $[M+H]^+$= calc. $C_{51}H_{71}N_5O_{13}SH^+$, 994.4847; found 994.4822. MS-ESI *m/z* (%): 994.47 (14) $[M+H]^+$; 1016.45 (8) $[M+Na]^+$. Melting point: 296 °C (dec.). R_f: 0.15 (CH_2Cl_2/MeOH, 9:1 v/v).

Adamantyl triacid 9: Triester **1** [1] (1.00 g; 2.24 mmol) was dissolved in THF (400 mL) and treated with EDC·HCl (865 mg; 4.52 mmol), DMAP (55 mg; 45 µmol) and 1-(2-carboxyethyl)adamantane [3] (416 mg; 1.99 mmol). The resulting solution was stirred for 72 h at rt and the solvent was evaporated under reduced pressure. The resulting residue was dissolved in 2 M aqueous HCl (50 mL) and extracted three times with CH_2Cl_2 (50 mL each). The combined organics were washed two times with sat. aqueous $KHSO_4$ (50 mL each) and water (50 mL). The organic layer was dried over Na_2SO_4, filtered and the solvent was evaporated in vacuo. The remaining solid was purified by flash chromatography on silica (CH_2Cl_2) to give the intermediate adamantyl triester (1.15 g; 1.92 mmol; 96%) as a colorless solid. **^{1}H-NMR** (300 MHz, $CDCl_3$): δ = 5.16 (s, 1H, NH); 3.64 (s, 9H, 15-H); 2.24 (t, 6H, $^3J_{H,H}$ = 8.0 Hz, 6-H); 2.03 - 1.98 (m, 2H, 9-H); 1.92 (s, 3H; 13-H); 1.74 (d, 3H, $^2J_{H,H}$ = 12.1 Hz, 12a-H); 1.63 - 1.56 (m, 9H, 2-H, 12b-H); 1.54 (t, 6H, $^3J_{H,H}$ = 8.0 Hz, 5-H); 1.43 (s, 6H, 14-H); 1.36 - 1.30 (m, 2H, 10-H); 1.13 (d, 3H, $^2J_{H,H}$ = 11.9 Hz, 4a-H); 1.02 (d, 3H, $^2J_{H,H}$ = 11.9 Hz, 4b-H). **^{13}C-NMR** (75 MHz, $CDCl_3$): δ = 174.5 (C7); 173.6 (C8); 53.7 (C1); 51.7 (C11); 45.1 (C4); 44.8 (C2); 42.2 (C13); 39.9 (C3); 37.5 (C12); 37.1 (C14); 34.9 (C15); 32.0 (C5); 31.2 (C10); 28.7 (C6); 28.1 (C9). HRMS-ESI: *m/z* $[M+H]^+$= calc. $C_{35}H_{53}NO_7H^+$, 600.3900; found 600.3904. HRMS-ESI: *m/z* $[M+Na]^+$= calc. $C_{35}H_{53}NO_7Na^+$,

622.3720; found 622.3722. CHN: calc. $C_{35}H_{53}NO_7$ C: 70.09, H: 8.91, N: 2.34; found C: 69.30, H: 8.85, N: 1.94. Melting point: 101 °C. R_f: 0.20 (CH_2Cl_2).

This intermediate (552 mg; 0.921 mmol) was dissolved in THF (80 mL), treated with KOTMS (potassium trimethylsilanolate; 1.06 g; 8.29 mmol) and stirred for 20 h at rt. The solvent was evaporated in vacuo and the residue was dissolved in 2 M aqueous HCl and extracted four times with EtOAc (50 mL each). The combined organics were dried over Na_2SO_4, filtered and the solvent was evaporated. The title compound **9** was obtained as a colorless solid (483 mg; 867 µmol; 94%). **^{1}H-NMR** (300 MHz, Methanol-d_4): δ = 7.46 (s, 1H, NH); 2.27 (t, 6H, $^3J_{H,H}$ = 8.0 Hz, 6-H); 2.10 - 2.05 (m, 2H, 9-H); 1.95 (s, 3H; 13-H); 1.75 (d, 3H, $^2J_{H,H}$ = 12.3 Hz, 12a-H); 1.69 - 1.64 (m, 9H, 2-H, 12b-H); 1.55 - 1.51 (m, 12H, 5-H, 14-H); 1.37 - 1.31 (m, 2H, 10-H); 1.19 (d, 3H, $^2J_{H,H}$ = 12.2 Hz, 4a-H); 1.12 (d, 3H, $^2J_{H,H}$ = 12.2 Hz, 4b-H). **^{13}C-NMR** (75 MHz, Methanol-d_4): δ = 178.1 (C7); 176.9 (C8); 54.6 (C1); 46.2 (C4); 45.5 (C2); 43.2 (C12); 41.5 (C11); 39.0 (C3); 38.2 (C13); 35.8 (C14); 33.1 (C5); 31.7 (C10); 30.1 (C9); 29.0 (C6). HRMS-ESI: *m/z* $[M+H]^+$= calc. $C_{32}H_{47}NO_7H^+$, 558.3431; found 558.3424. HRMS-ESI: *m/z* $[M+Na]^+$= calc. $C_{32}H_{47}NO_7Na^+$, 580.3250; found 580.3242. CHN: calc. $C_{32}H_{47}NO_7$ C: 68.91, H: 8.49, N: 2.51; found C: 69.00, H: 8.44, N: 2.47. Melting point: 123 °C. R_f: 0.25 (CH_2Cl_2/MeOH, 9:1 v/v).

Adamantyl triscatecholate 10: Adamantyl triacid (100 mg; 179 µmol) was dissolved in DMF (30 mL) and DIEA (*N*,*N*-diisopropylethylamine, 49.0 µL, 286 µmol) were added at 0 °C. The solution was treated with EDC·HCl (113 mg; 591 µmol) and HOBt (80.0 mg; 591 µmol) in DMF (5 mL each). The resulting solution was stirred for 30 min at 0 °C and dopamine hydrochloride (112 mg; 591 µmol) in DMF (10 mL) were added. The solution was stirred for 72 h at rt and the solvent was evaporated under reduced pressure. The resulting residue was washed two times with 2 M aqueous HCl (50 mL each) and two times with EtOAc (50 mL each). Freeze drying

gave the title compound (161 mg; 167 µmol; 93%) as a colorless solid. **^{1}H-NMR** (300 MHz, Methanol-d_4): δ = 6.68 (d, 3H, $^3J_{H,H}$ = 8.0 Hz, 21-H); 6.64 (d, 3H, $^4J_{H,H}$ = 1.9 Hz, 18-H); 6.52 (dd, 3H, $^3J_{H,H}$ = 8.0 Hz, $^4J_{H,H}$ = 1.9 Hz, 22-H); 3.37 - 3.30 (m, 6H, 15-H); 2.64 (t, 6H, $^3J_{H,H}$ = 7.1 Hz, 16-H); 2.18 - 2.06 (m, 8H, 6-H, 9-H); 1.93 (s, 3H, 13-H); 1.74 (d, 3H, $^2J_{H,H}$ = 12.5 Hz, 12a-H); 1.67 - 1.61 (m, 9H, 2-H, 12b-H); 1.47 - 1.41 (m, 12H, 4-H, 14-H); 1.34 - 1.29 (m, 2H, 10-H); 1.15 (d, 3H, $^2J_{H,H}$ = 12.6 Hz 4a-H); 1.05 (d, 3H, $^2J_{H,H}$ = 12.6 Hz 4b-H). **^{13}C-NMR** (75 MHz, Methanol-d_4): δ = 177.0 (C7); 176.9 (C8); 146.2 (C19); 144.8 (C20); 131.8 (C17); 121.0 (C18); 116.9 (C21); 116.3 (C22); 55.1 (C1); 46.2 (C4); 45.4 (C2); 43.2 (C12); 42.4 (C14); 41.6 (C13); 40.1 (C15); 38.1 (C5); 36.1 (C11); 35.7 (C16); 34.5 (C3); 31.6 (C10); 30.9 (C9); 30.1 (C6). HRMS-ESI: *m/z* $[M+H]^+$= calc. $C_{56}H_{74}N_4O_{10}H^+$, 963.5483; found 963.5499. Melting point: 143 °C. R_f: 0.10 (CH_2Cl_2/MeOH, 9:1 v/v).

Acrylamido triscatecholate 11: Acrylamide **4** (1.17 g; 2.52 mmol) was dissolved in THF (80 mL), treated with KOTMS (potassium trimethylsilanolate; 4.85 g; 37.8 mmol) and stirred for 17 h at rt. The solvent was evaporated in vacuo and the residue was dissolved in 1 M aqueous HCl and extracted four times with EtOAc (50 mL each). The combined organics were dried over Na_2SO_4, filtered and the solvent was evaporated. The intermediate triacid was obtained as an orange solid (1.02 g; 2.42 mmol; 96%). **^{1}H-NMR** (300 MHz, DMSO-d_6): δ = 12.02 (s, 3H, COOH); 7.61 (s, 1H, NH); 6.23 (dd, 1H, $^3J_{H,H}$ = 10.0 Hz, $^3J_{H,H}$ = 16.9 Hz, 9-H); 5.99 (dd, 1H, $^2J_{H,H}$ = 2.2 Hz, $^3J_{H,H}$ = 16.9 Hz, 10[E to 9]-H); 5.48 (dd, 1H, $^2J_{H,H}$ = 2.2 Hz, $^3J_{H,H}$ = 10.0 Hz, 10[Z to 9]-H); 2.15 (t, 6H, $^3J_{H,H}$ = 7.8 Hz, 6-H); 1.55 (s, 6H, 2-H); 1.39 (t, 6H, $^3J_{H,H}$ = 7.8 Hz, 5-H); 1.10 - 0.99 (m, 6H, 4-H). **^{13}C-NMR** (75 MHz, DMSO-d_6): δ = 175.0 (C7); 163.8 (C8); 132.8 (C10); 124.3 (C9); 52.9 (C1); 44.6 (C4); 44.3 (C2); 37.4 (C5); 34.3 (C3); 27.9 (C6). HRMS-ESI: *m/z* $[M+H]^+$= calc. $C_{22}H_{31}NO_7H^+$, 422.2173; found 422.2162. CHN: calc.

$C_{22}H_{31}NO_7$ C: 62.69, H: 7.41, N: 3.32; found C: 61.42, H: 7.22, N: 3.19. Melting point: 129 °C. R_f: 0.10 (CH_2Cl_2/MeOH; 9:1; v/v).

This intermediate (240 mg; 570 µmol) was dissolved in DMF (20 mL) and DIEA (*N*,*N*-diisopropylethylamine, 0.16 mL, 0.91 mmol) were added at 0 °C. The solution was treated with EDC·HCl (360 mg; 1.88 mmol) and HOBt (250 mg; 1.88 mmol) in DMF (5 mL each). The resulting solution was stirred for 30 min at 0 °C and dopamine hydrochloride (360 mg; 1.88 mmol) in DMF (5 mL) were added. The mixture was stirred for 72 h at rt and the solvent was removed in vacuo. The resulting residue was dissolved in 2 M aqueous HCl (50 mL) and extracted three times with EtOAc (50 mL each). The combined organics were washed three times with sat. aqueous $KHSO_4$ (20 mL each), dried over Na_2SO_4, filtered and the solvent was evaporated. The crude product was suspended in 50 mL Et_2O and stirred at 30 °C for 30 min. The resulting slurry was filtered and the procedure repeated five times to give the title compound **11** (344 mg; 416 µmol; 73%) as a colorless solid. **^{1}H-NMR** (300 MHz, Methanol-d_4): δ = 6.68 (d, 3H, $^3J_{H,H}$ = 8.4 Hz, 17-H); 6.65 (d, 3H, $^4J_{H,H}$ = 1.9 Hz, 14-H); 6.52 (dd, 3H, $^3J_{H,H}$ = 8.4 Hz, $^4J_{H,H}$ = 1.9 Hz, 18-H); 6.25 (dd, 1H, $^3J_{H,H}$ = 10.5 Hz, ^3J = 16.9 Hz, 9-H); 6.13 (dd, 1H, $^2J_{H,H}$ = 2.2 Hz, $^3J_{H,H}$ = 16.9 Hz, 10[E to 9]-H); 5.56 (dd, 1H, $^2J_{H,H}$ = 2.2 Hz, $^3J_{H,H}$ = 10.5 Hz, 10[Z to 9]-H); 3.37 - 3.32 (m, 6H, 11-H); 2.64 (t, 6H, $^3J_{H,H}$ = 7.4 Hz, 10-H); 2.17 - 2.12 (m, 6H, 6-H); 1.64 (s, 6H, 2-H); 1.51 - 1.44 (m, 6H, 5-H); 1.15 - 1.06 (m, 6H, 4-H). **^{13}C-NMR** (75 MHz, Methanol-d_4): δ = 177.0 (C7); 167.3 (C8); 146.1 (C15); 144.7 (C16); 133.1 (C10); 131.8 (C13); 125.9 (C9); 121.0 (C14); 116.9 (C17); 116.3 (C18); 55.0 (C1); 46.1 (C4); 45.4 (C2); 42.0 (C11); 40.0 (C5); 36.0 (C12); 35.7 (C3); 31.0 (C6). HRMS-ESI: *m/z* $[M+H]^+$= calc. $C_{46}H_{58}N_4O_{10}H^+$, 827.4231; found 827.4226. HRMS-ESI: *m/z* $[M+Na]^+$= calc. $C_{46}H_{58}N_4O_{10}Na^+$, 849.4051; found 849.4045. Melting point: 97 °C. R_f: 0.15 (EtOAc/EtOH; 9:1; v/v).

Bromo triscatecholate 13: Bromo triacid **12** [4] (552 mg; 1.28 mmol) was dissolved in DMF (50 mL) and DIEA (*N*,*N*-diisopropylethylamine, 350 µL, 2.05 mmol) was added at 0 °C. The solution was treated with EDC·HCl (809 mg; 4.22 mmol) and HOBt (570 mg; 4.22 mmol) in DMF (10 mL each). The resulting solution was stirred for 30 min at 0 °C and dopamine hydrochloride (800 mg; 4.22 mmol), in DMF (10 mL) was added. The mixture was stirred for 72 h at rt and the solvent was removed in vacuo. The resulting residue was dissolved in EtOAc (200 mL) and washed three times with 1 M aqueous HCl (40 mL each), three times with sat. aqueous $KHSO_4$ (100 mL each) and brine (50 mL). The organic layer was dried over Na_2SO_4, filtered and the solvent was evaporated in vacuo. The crude product was suspended in 50 mL Et_2O and stirred at 30 °C for 30 min. The resulting slurry was filtered and the procedure repeated five times to give the title compound (761 mg; 909 µmol; 71%) as a colorless solid. **^{1}H-NMR** (300 MHz, Methanol-d_4): δ = 6.68 (d, 3H, $^3J_{H,H}$ = 8.1 Hz, 14-H); 6.64 (d, 3H, $^4J_{H,H}$ = 1.9 Hz, 11-H); 6.52 (dd, 3H, $^3J_{H,H}$ = 8.1 Hz, $^4J_{H,H}$ = 1.9 Hz, 15-H); 3.35 (s, 6H, 8-H); 2.63 (t, 6H, $^3J_{H,H}$ = 7.3 Hz, 9-H); 2.11 (t, 6H, $^3J_{H,H}$ = 8.1 Hz, 6-H); 1.93 (s, 6H, 2-H); 1.45 (t, 6H, $^3J_{H,H}$ = 8.1 Hz, 5-H); 1.19 (d, 3H, $^2J_{H,H}$ = 12.6 Hz, 4a-H); 1.10 (d, 3H, $^2J_{H,H}$ = 12.6 Hz, 4b-H). **^{13}C-NMR** (75 MHz, Methanol-d_4): δ = 176.5 (C7); 146.2 (C12); 144.7 (C13); 131.9 (C10); 121.0 (C11); 116.9 (C14); 116.3 (C15); 66.9 (C4); 66.1 (C2); 53.5 (C1); 42.2 (C5); 39.7 (C6); 38.9 (C8); 35.8 (C9); 31.0 (C3). HRMS-ESI: *m/z* $[MH]^+$= calc. $C_{43}H_{55}BrN_3O_9^+$, 838.3101; found 838.3096. IR: v /cm^{-1}= 3276; 2926; 2848; 1625; 1520; 1444; 1247; 1193; 959; 813. CHN: calc. $C_{43}H_{54}BrN_3O_9$ C: 61.72, H: 6.50, N: 5.02; found C: 60.96, H: 6.61, N: 4.94. Melting point: 154 °C. R$_f$: 0.25 (EtOAc/EtOH; 9:1; v/v).

NMR spectra

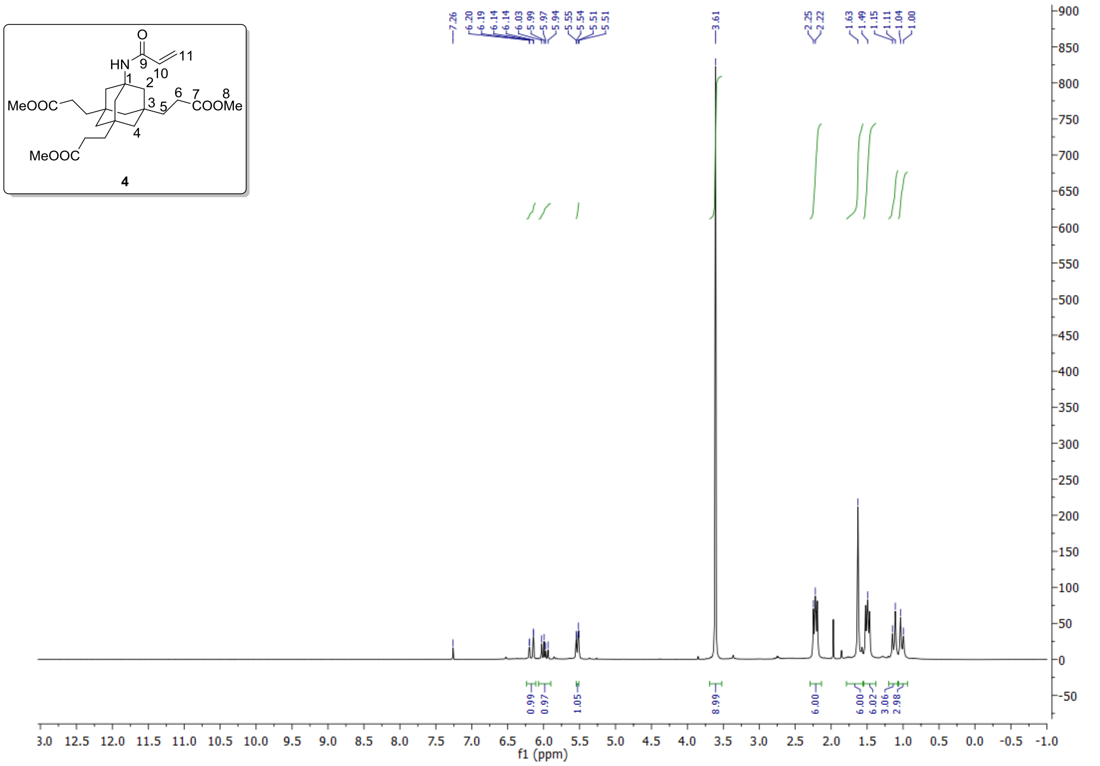

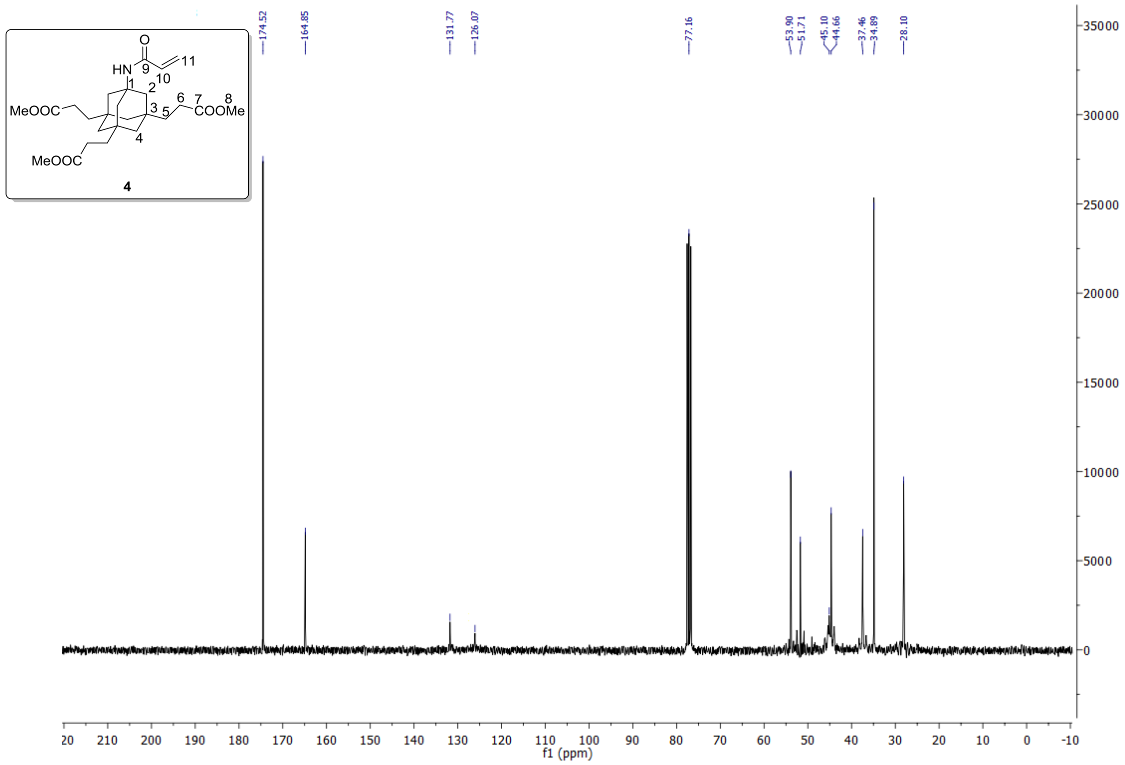

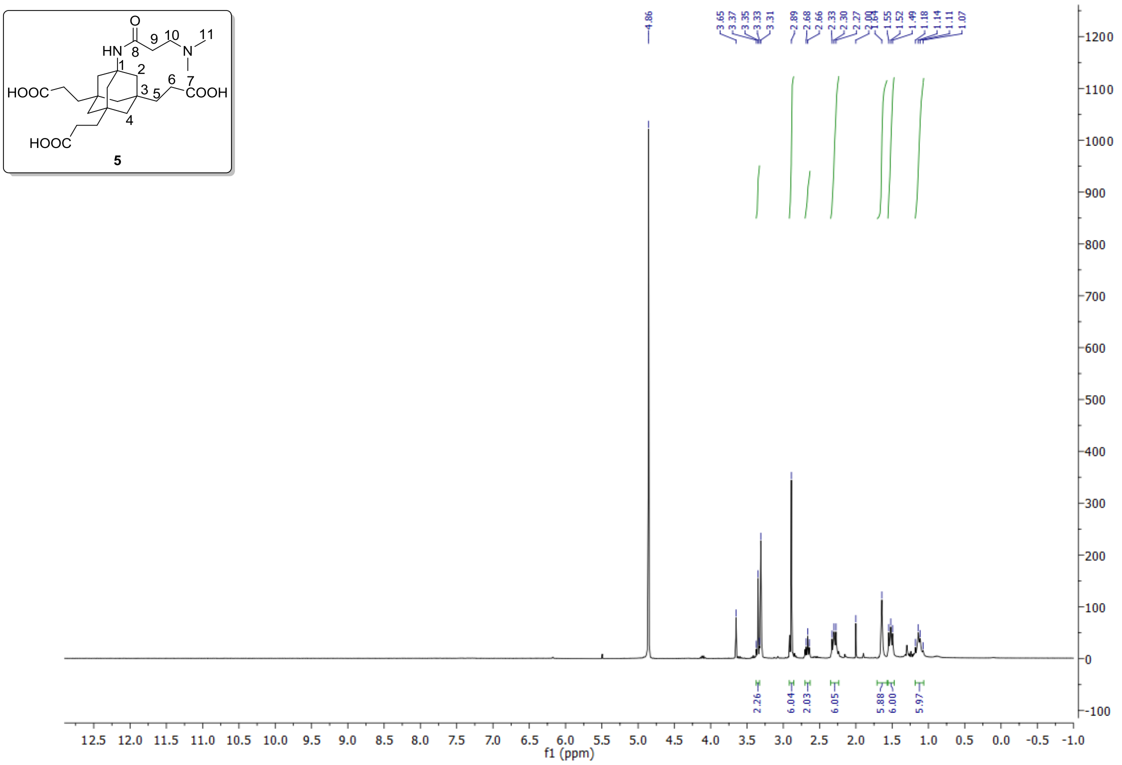
HOOC
HOOC
COOH
HN
N
O
5
4.86
3.65
3.37
3.35
3.33
3.31
2.89
2.68
2.66
2.33
2.30
2.27
1.55
1.52
1.49
1.18
1.14
1.11
1.07
2.26
6.04
2.03
6.05
5.88
6.00
5.97
f1 (ppm)

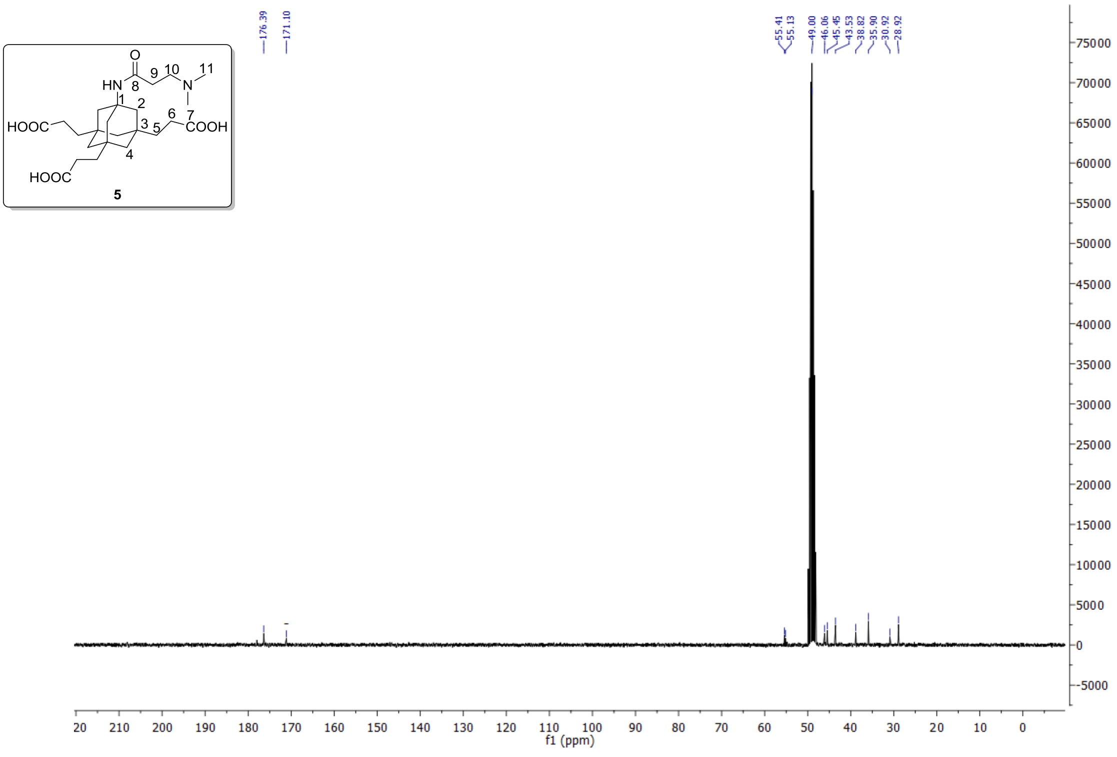
HOOC
HOOC
COOH
HN
N
O
5
176.39
171.10
55.41
55.13
49.00
46.06
45.46
43.53
38.82
35.90
30.92
28.92
f1 (ppm)

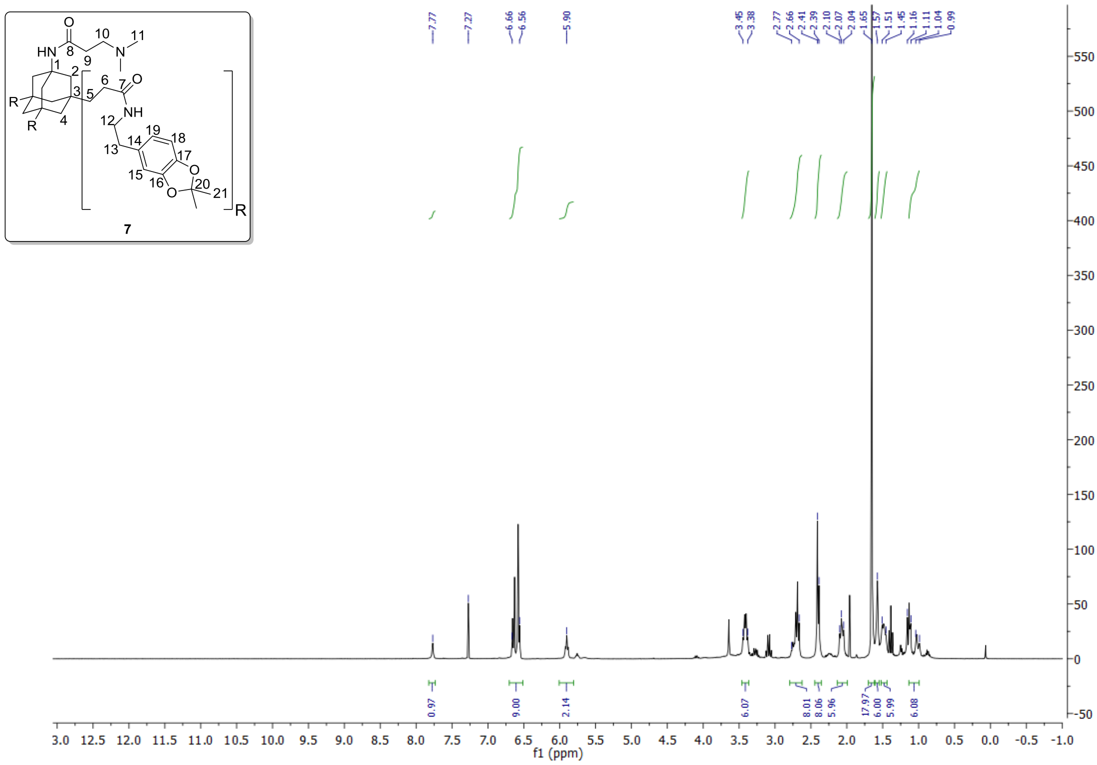
7
7.77
7.27
6.66
6.56
5.90
3.45
3.38
2.77
2.66
2.41
2.39
2.10
2.07
2.04
1.65
1.57
1.51
1.45
1.16
1.11
1.04
0.99
0.97
9.00
2.14
6.07
8.01
8.06
5.96
17.97
6.00
5.99
6.08
f1 (ppm)

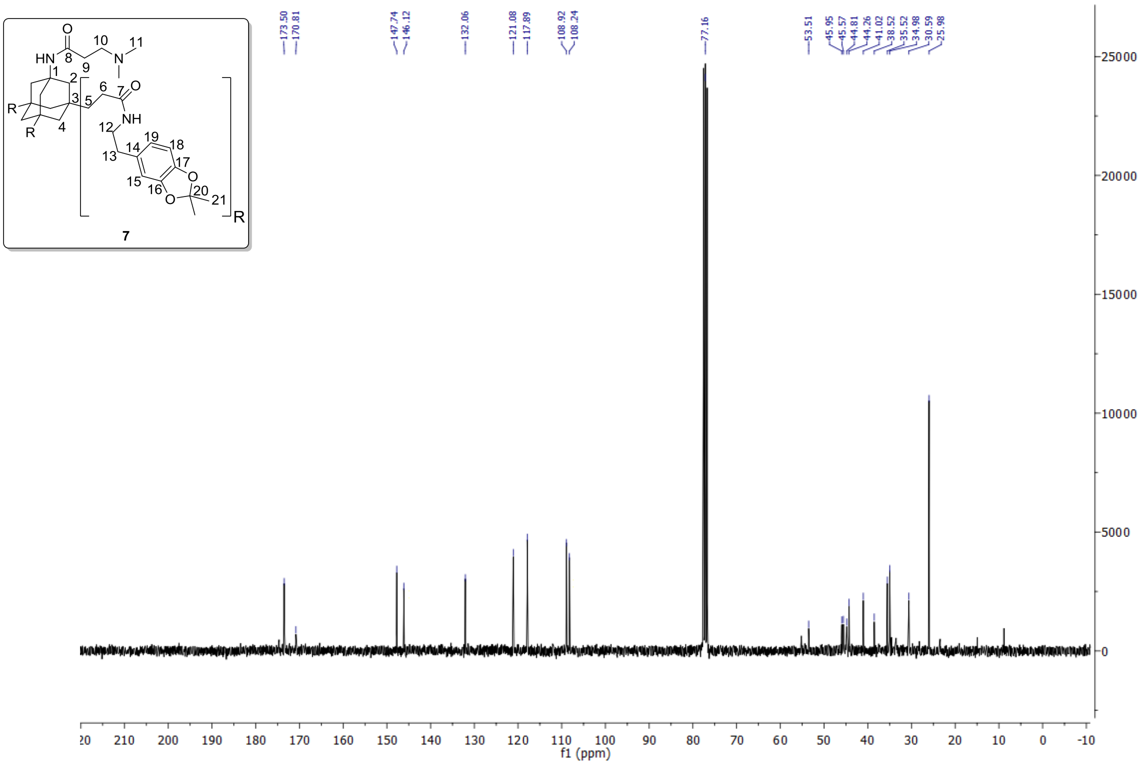
7
173.50
170.81
147.74
146.12
132.06
121.08
117.89
108.92
108.24
77.16
53.51
45.95
45.57
44.81
44.26
41.02
38.52
35.52
34.98
30.59
25.98
f1 (ppm)

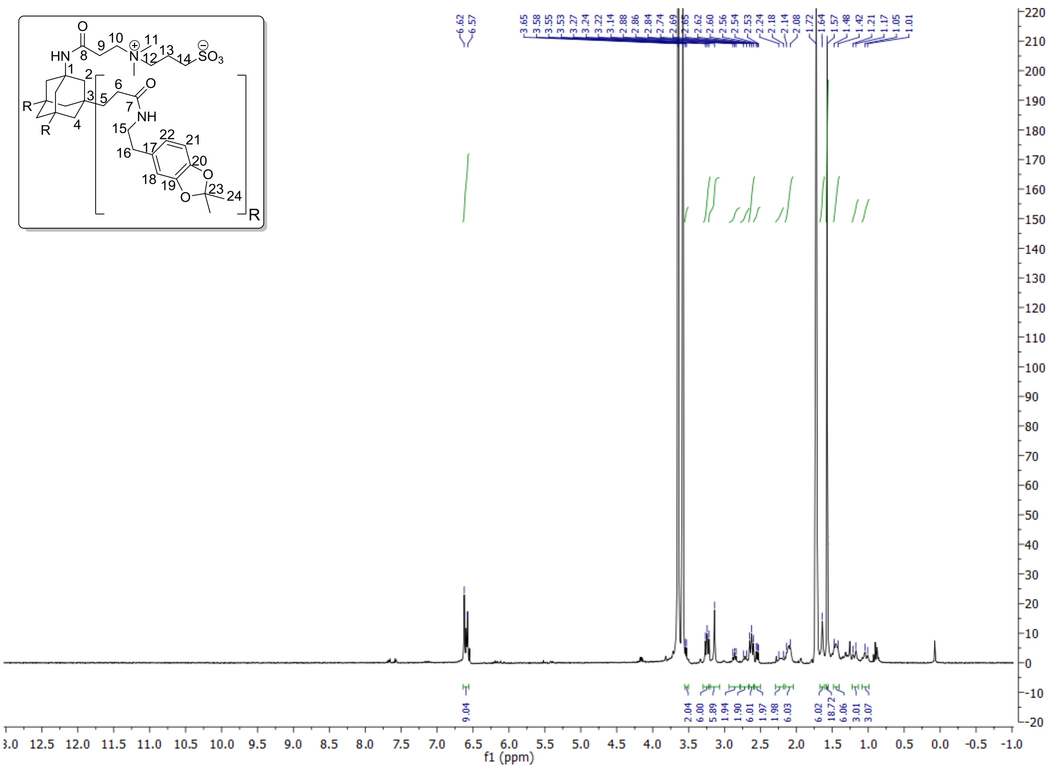
f1 (ppm)

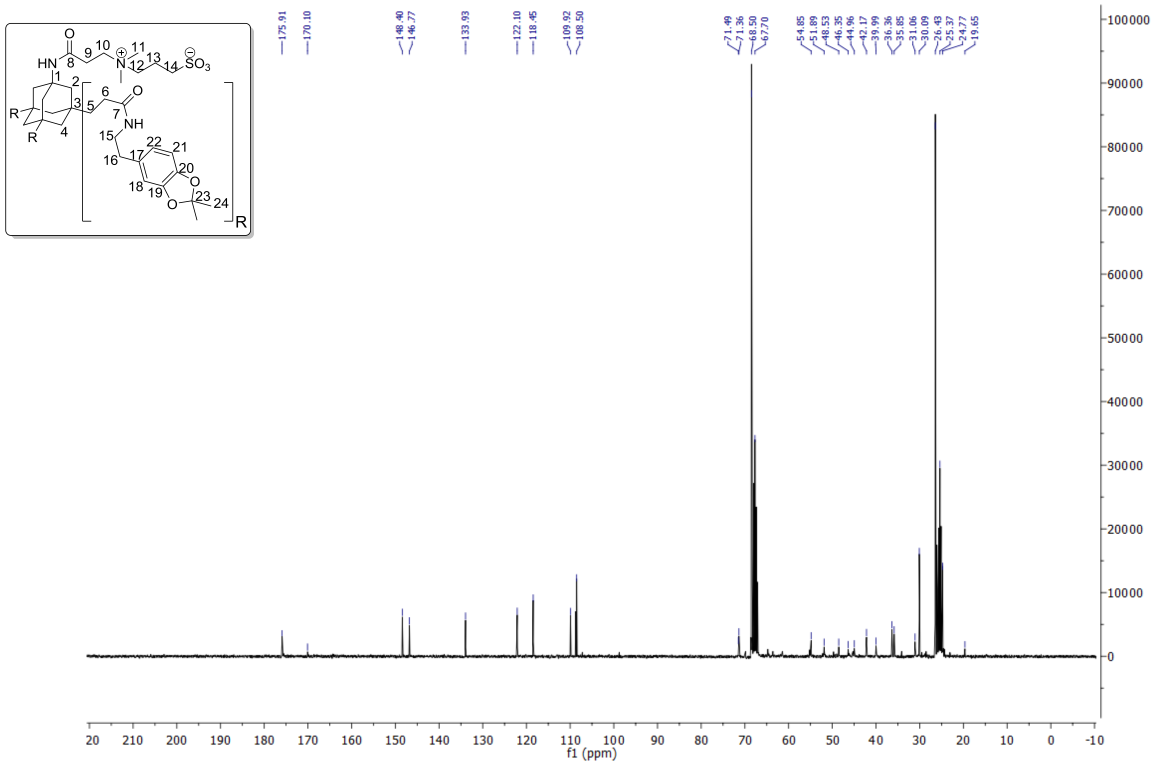
f1 (ppm)

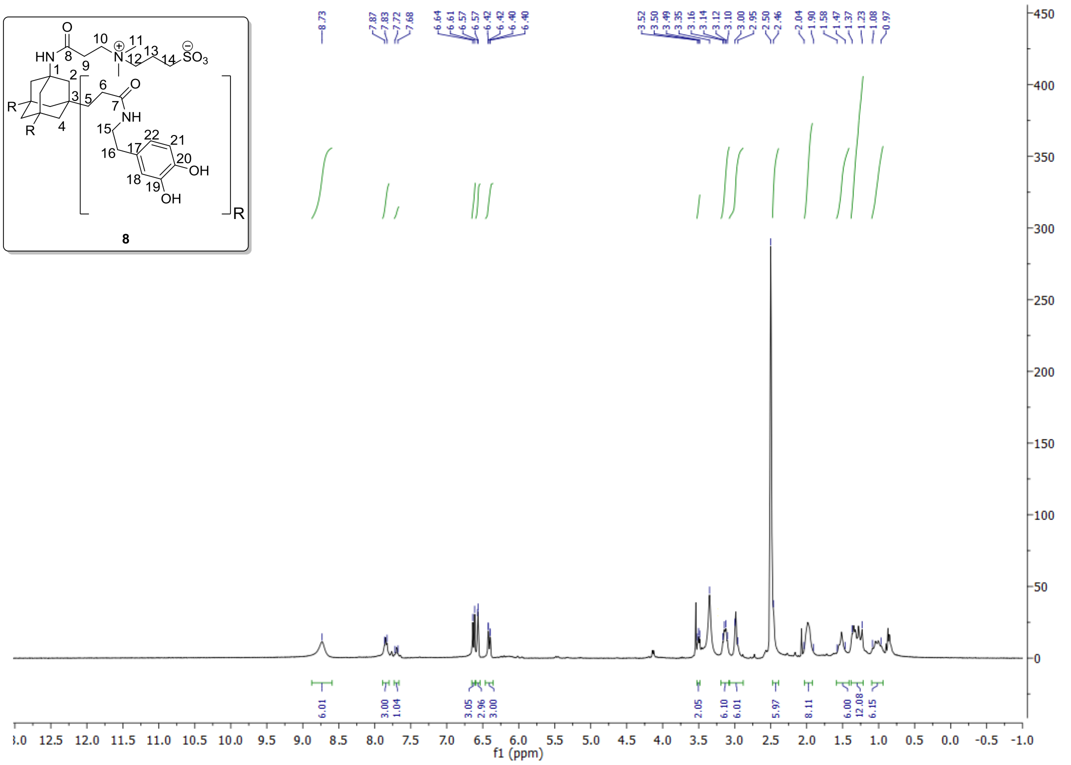

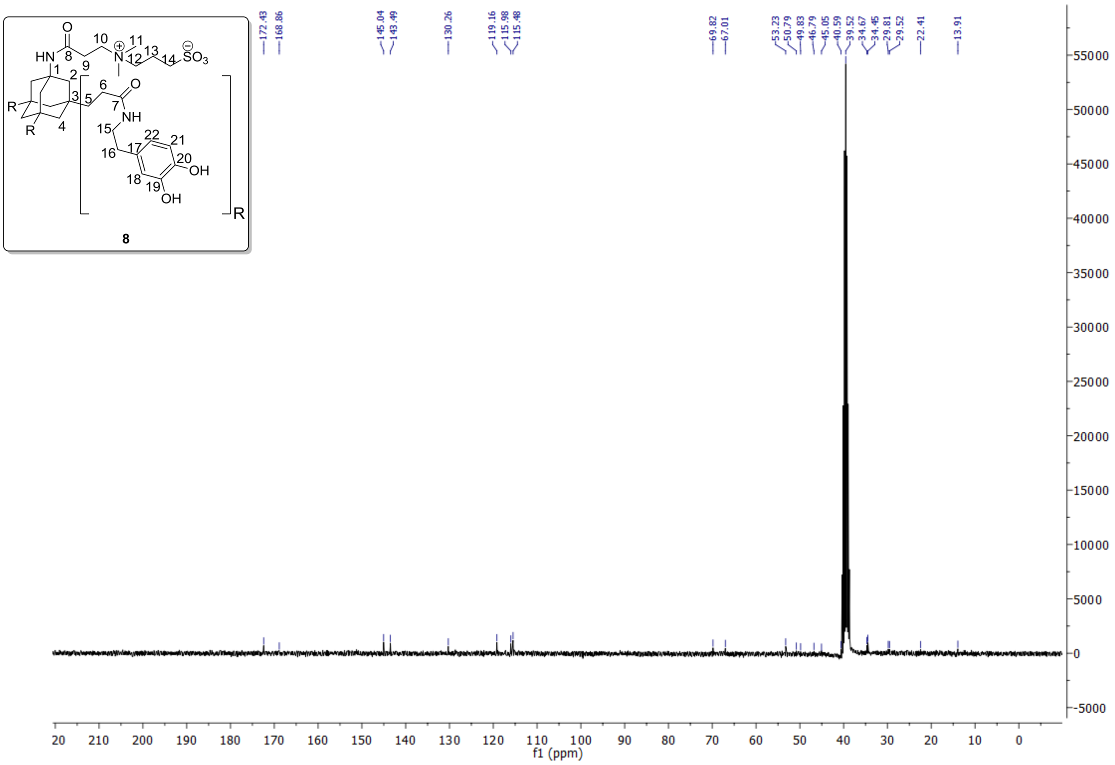

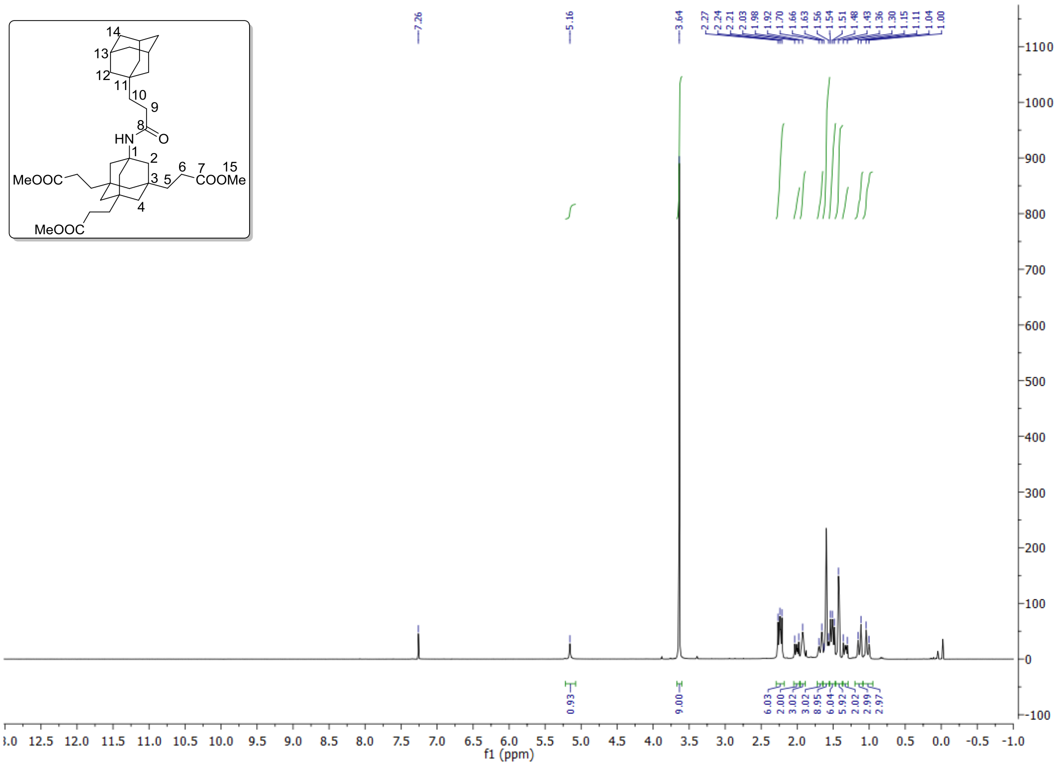
HN
O
MeOOC
COOMe
MeOOC
7.26
5.16
3.64
2.27
2.24
2.21
2.03
1.98
1.92
1.70
1.66
1.63
1.56
1.54
1.51
1.48
1.43
1.36
1.30
1.15
1.11
1.04
1.00
0.93
9.00
6.03
2.00
3.02
3.02
8.95
6.04
5.92
2.02
2.99
2.97
f1 (ppm)

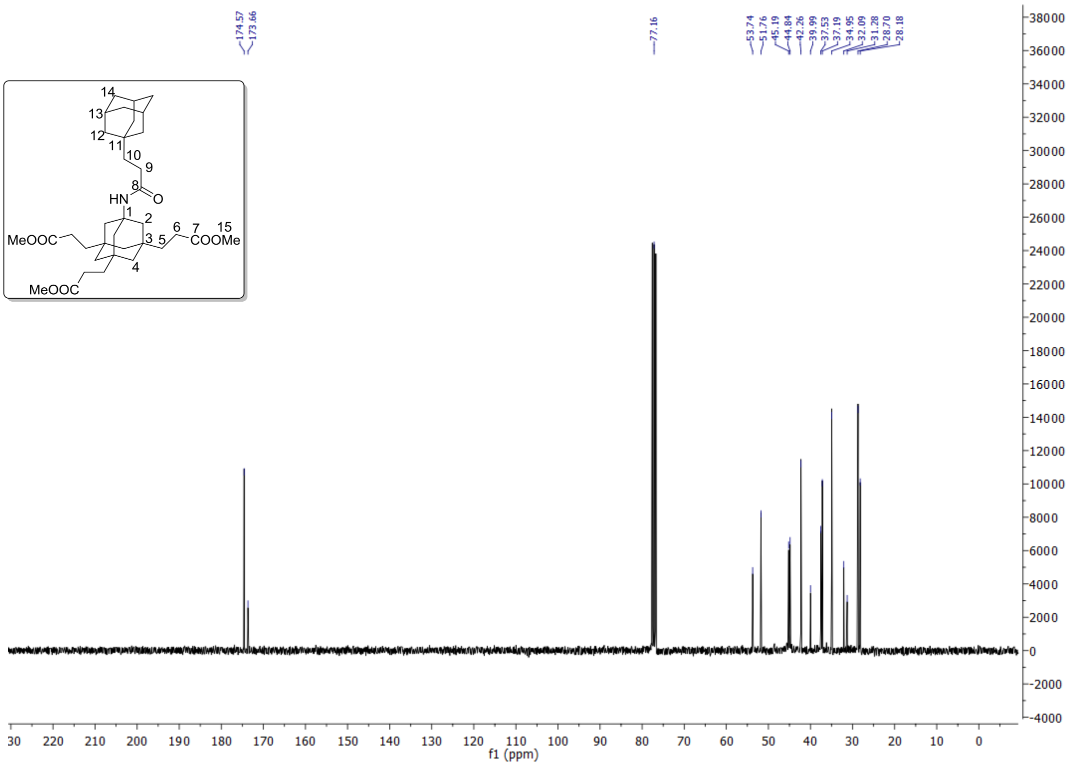
HN
O
MeOOC
COOMe
MeOOC
174.57
173.66
77.16
53.74
51.76
45.19
44.84
42.26
39.99
37.53
37.19
34.95
32.09
31.28
28.70
28.18
f1 (ppm)

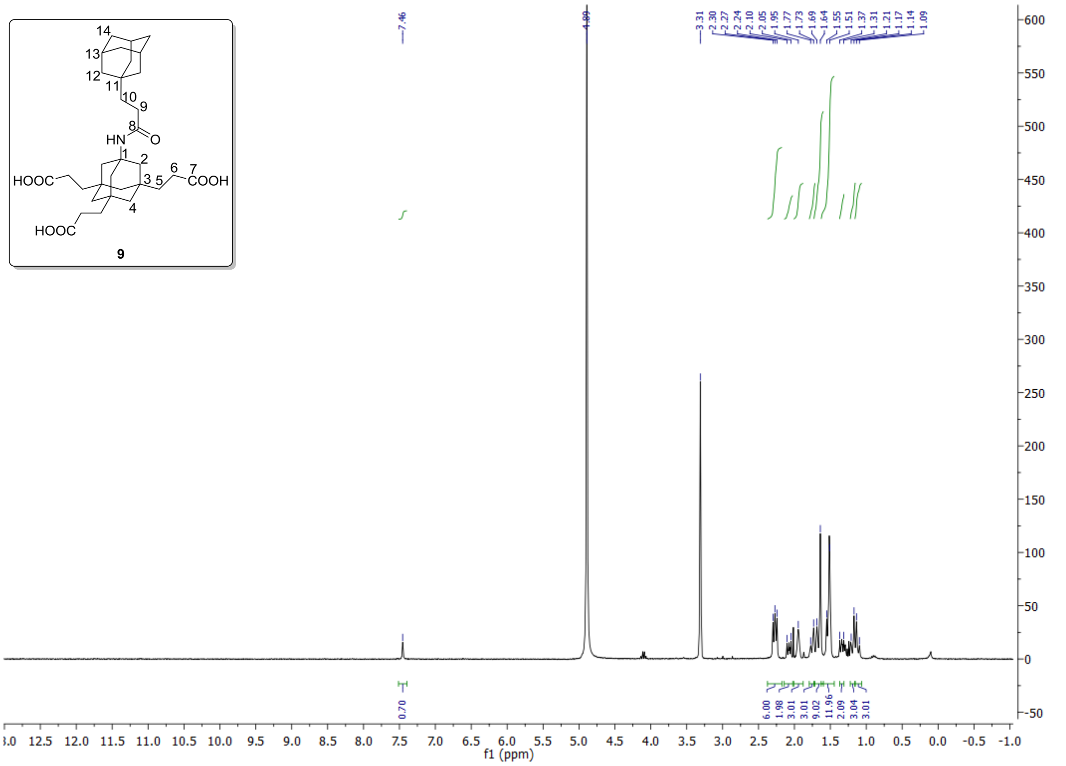

7.46
4.89
3.31
2.30
2.27
2.24
2.10
2.05
1.95
1.77
1.73
1.69
1.64
1.55
1.51
1.37
1.31
1.21
1.17
1.14
1.09
0.70
6.00
1.98
3.01
3.01
9.02
11.96
2.09
3.04
3.01
HOOC
COOH
HN
O
9
f1 (ppm)

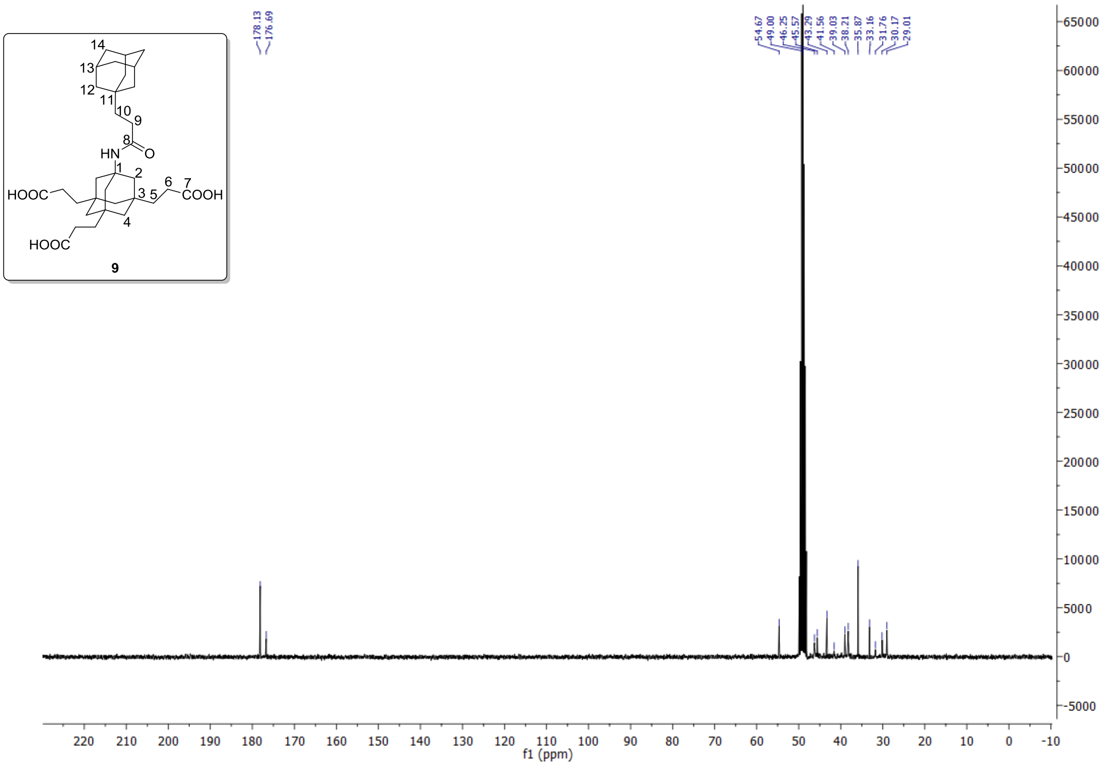

178.13
176.69
54.67
49.00
46.25
45.57
43.29
41.56
39.03
38.21
35.87
33.16
31.76
30.17
29.01
HOOC
COOH
HN
O
9
f1 (ppm)

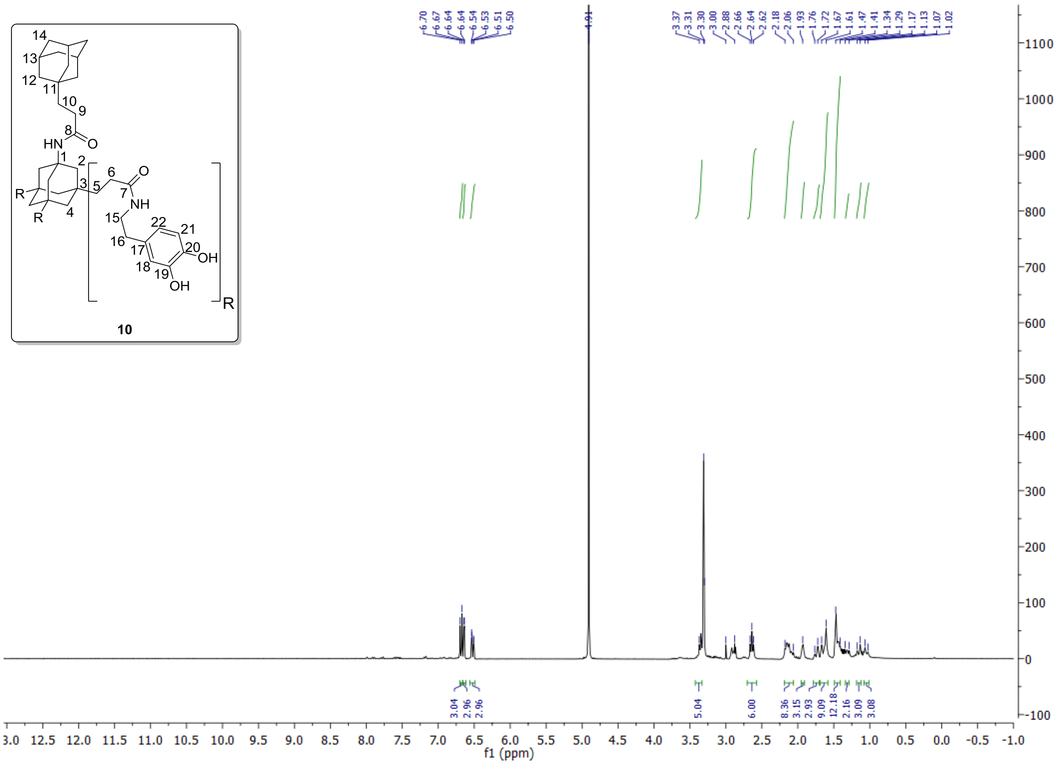
6.70
6.67
6.64
6.54
6.53
6.51
6.50
4.91
3.37
3.31
3.30
3.00
2.88
2.66
2.64
2.62
2.18
2.06
1.93
1.76
1.72
1.67
1.61
1.47
1.41
1.34
1.29
1.17
1.13
1.07
1.02
3.04
2.96
2.96
5.04
6.00
8.36
3.15
2.93
9.09
12.18
2.16
3.09
3.08
f1 (ppm)
10

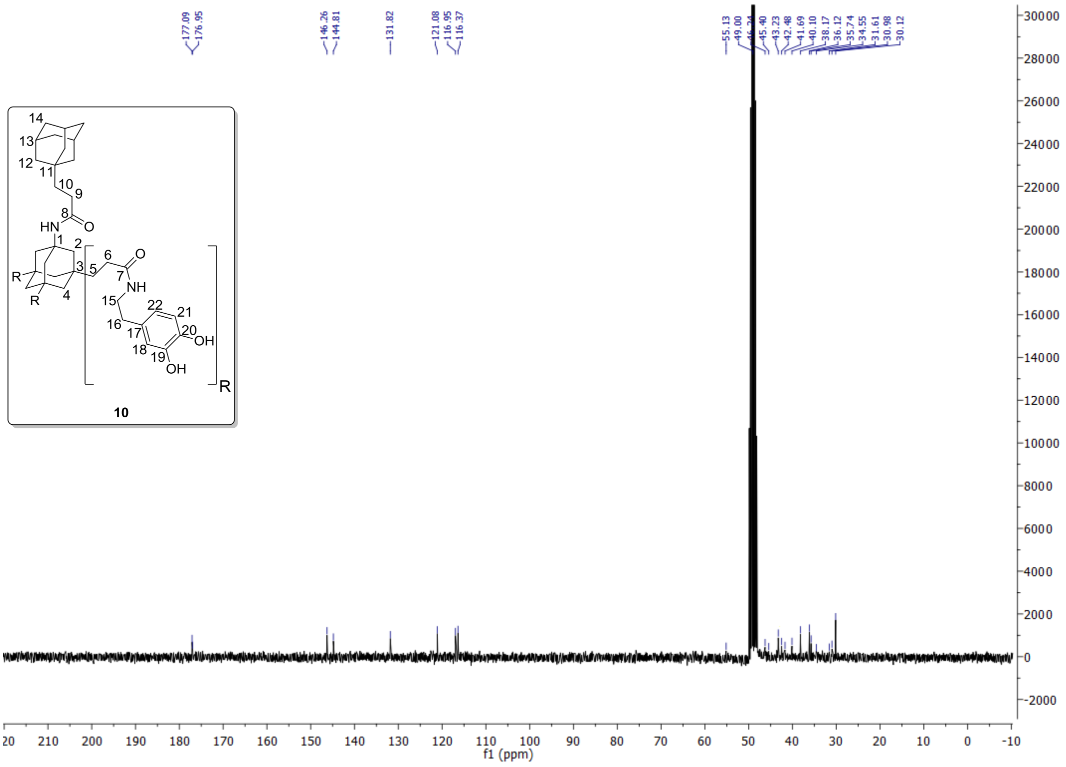
177.09
176.95
146.26
144.81
131.82
121.08
116.95
116.37
55.13
49.00
45.40
43.23
42.48
41.69
40.10
38.17
36.12
35.74
34.55
31.61
30.98
30.12
f1 (ppm)
10

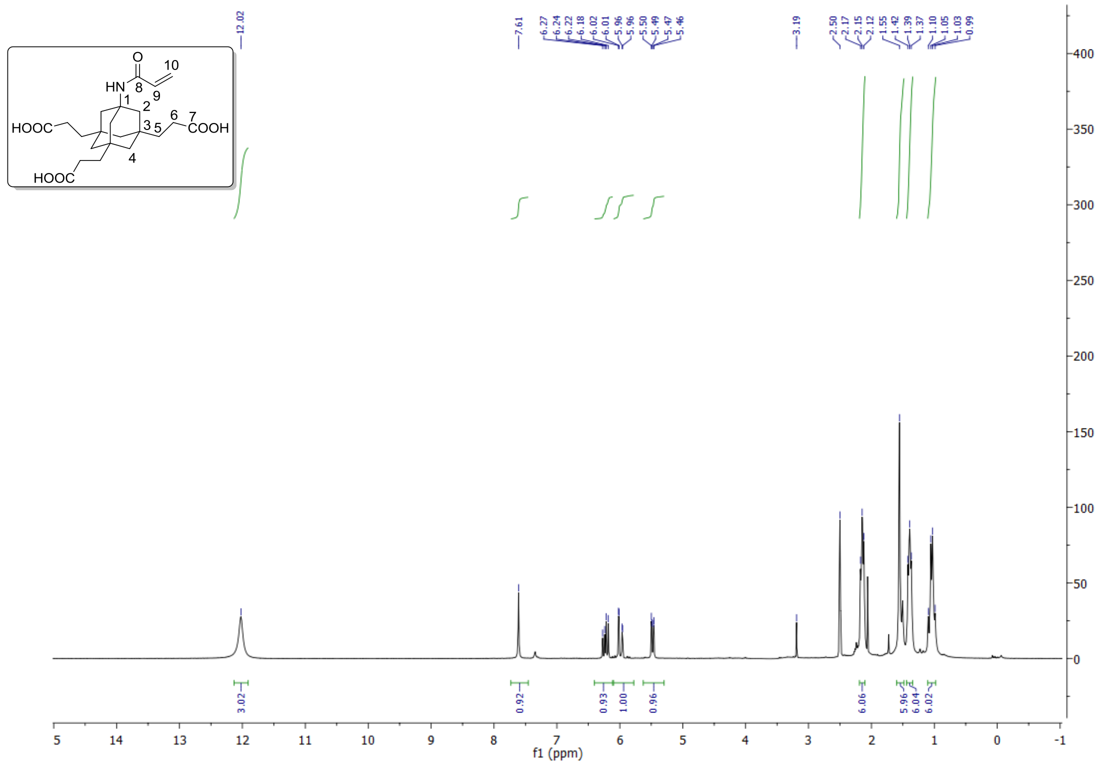
HOOC
HOOC
HOOC
COOH
HN
O
12.02
7.61
3.19
f1 (ppm)

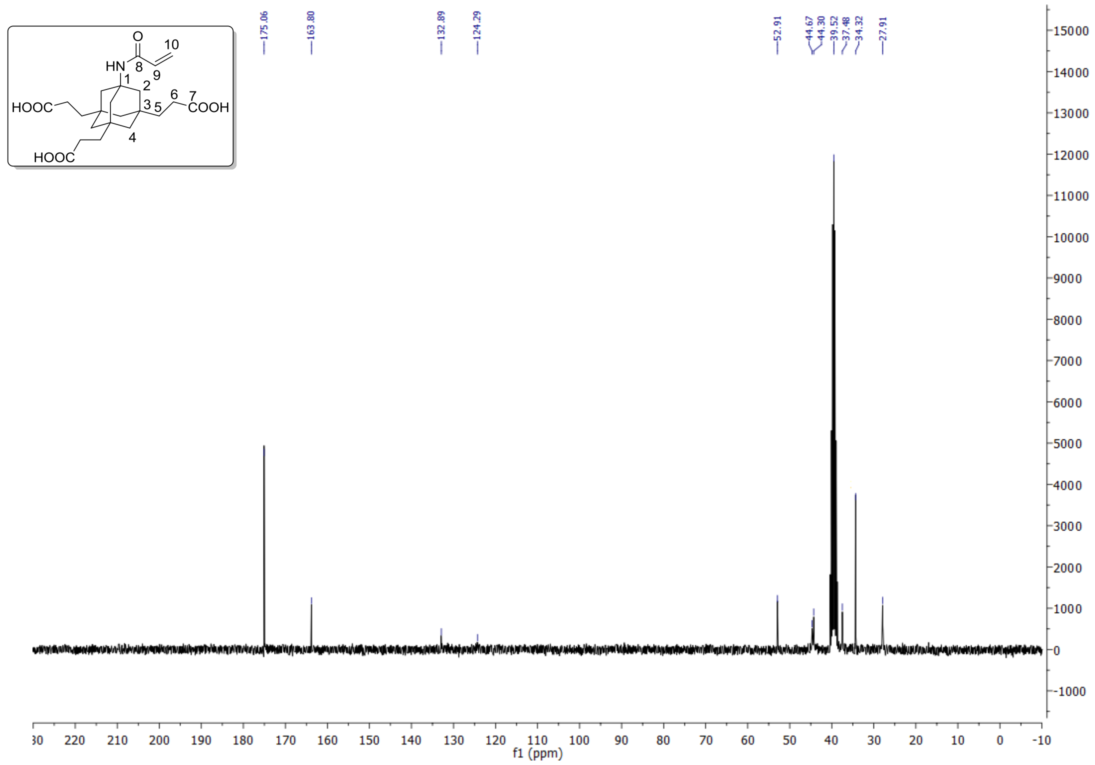
175.06
163.80
132.89
124.29
52.91
44.67
44.30
39.52
37.48
34.32
27.91
f1 (ppm)

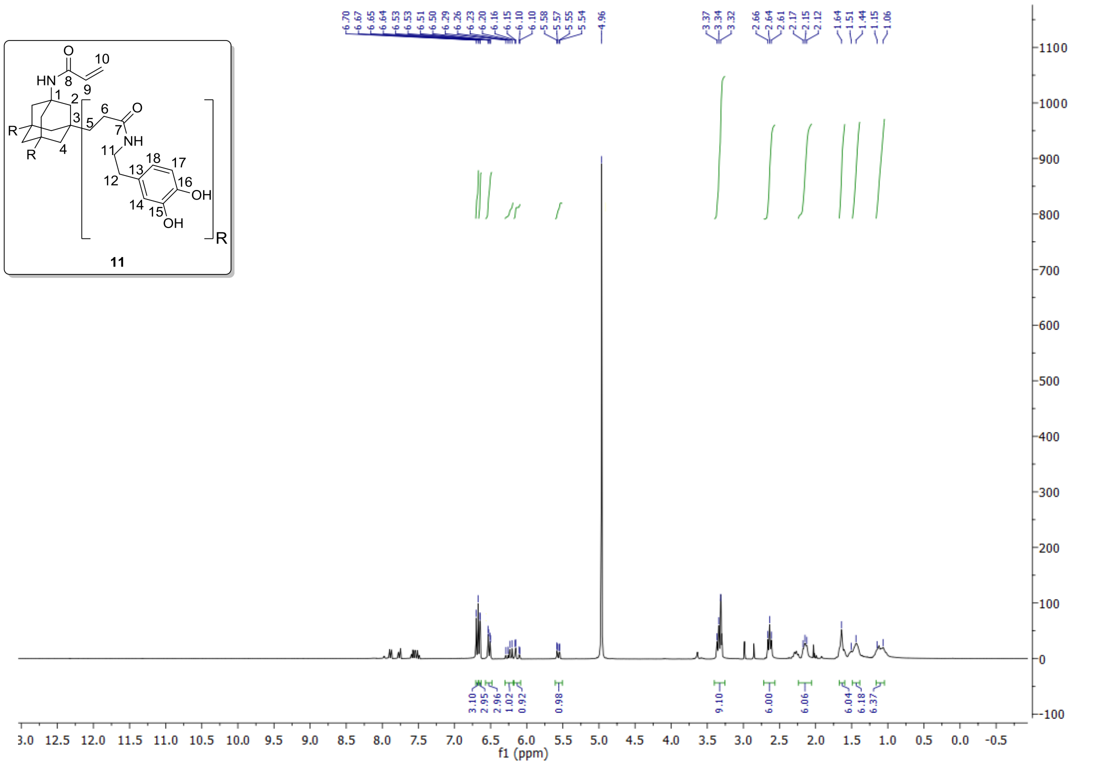
6.70
6.67
6.65
6.64
6.53
6.53
6.51
6.50
6.29
6.26
6.23
6.20
6.16
6.15
6.10
6.10
5.58
5.57
5.55
5.54
4.96
3.37
3.34
3.32
2.66
2.64
2.61
2.17
2.15
2.12
1.64
1.51
1.44
1.15
1.06
3.10
2.95
2.96
1.02
0.92
0.98
9.10
6.00
6.06
6.04
6.18
6.37
f1 (ppm)
O
10
HN
8
1
9
2
6
O
R
3
5
7
NH
R
4
11
13
18
17
12
16
OH
14
15
OH
R
11

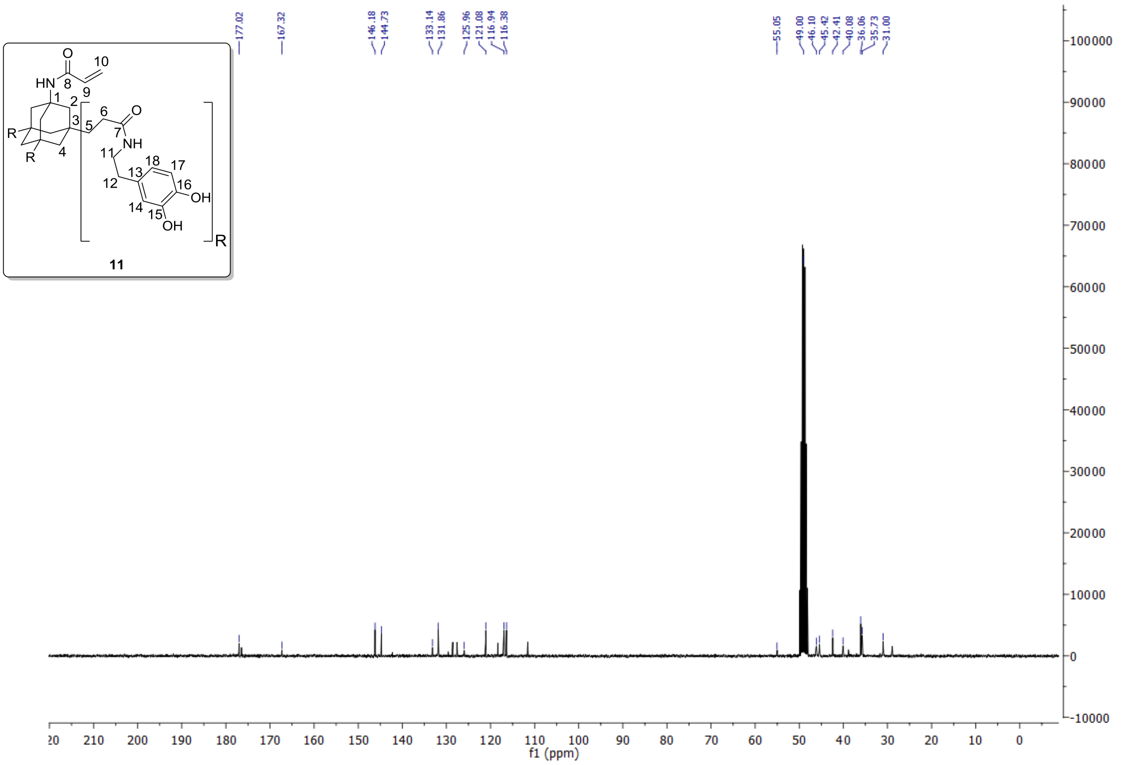
177.02
167.32
146.18
144.73
133.14
131.86
125.96
121.08
116.94
116.38
55.05
49.00
46.10
45.42
42.41
40.08
36.06
35.73
31.00
f1 (ppm)
O
10
HN
8
1
9
2
6
O
R
3
5
7
NH
R
4
11
13
18
17
12
16
OH
14
15
OH
R
11

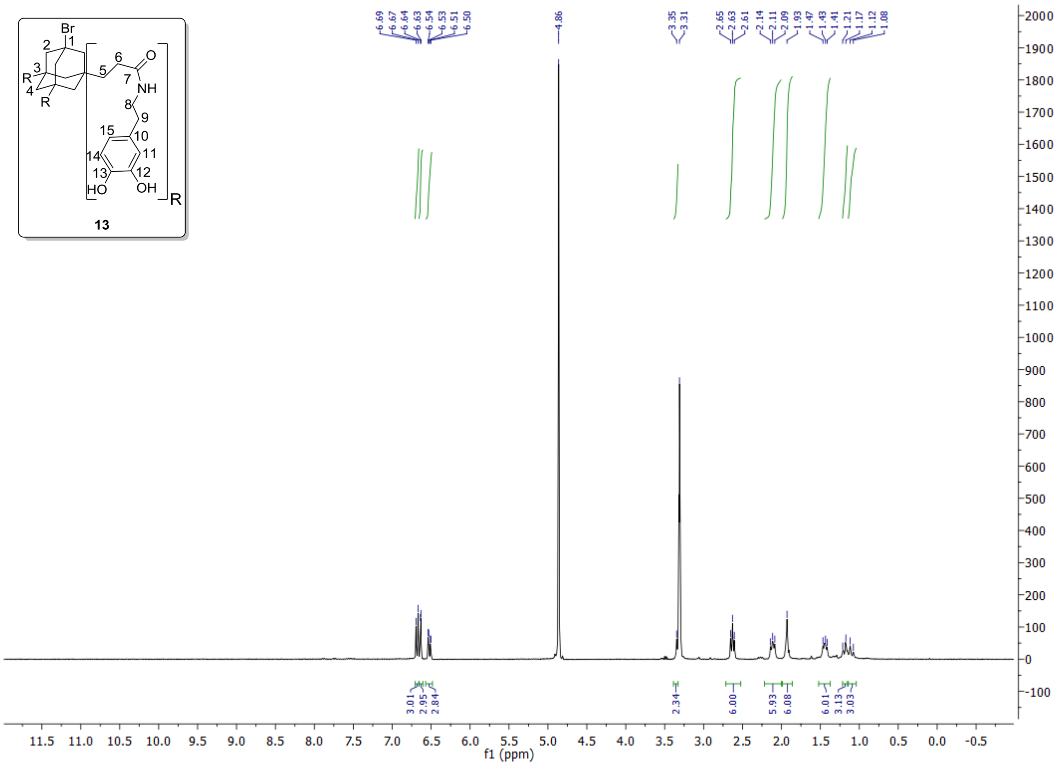

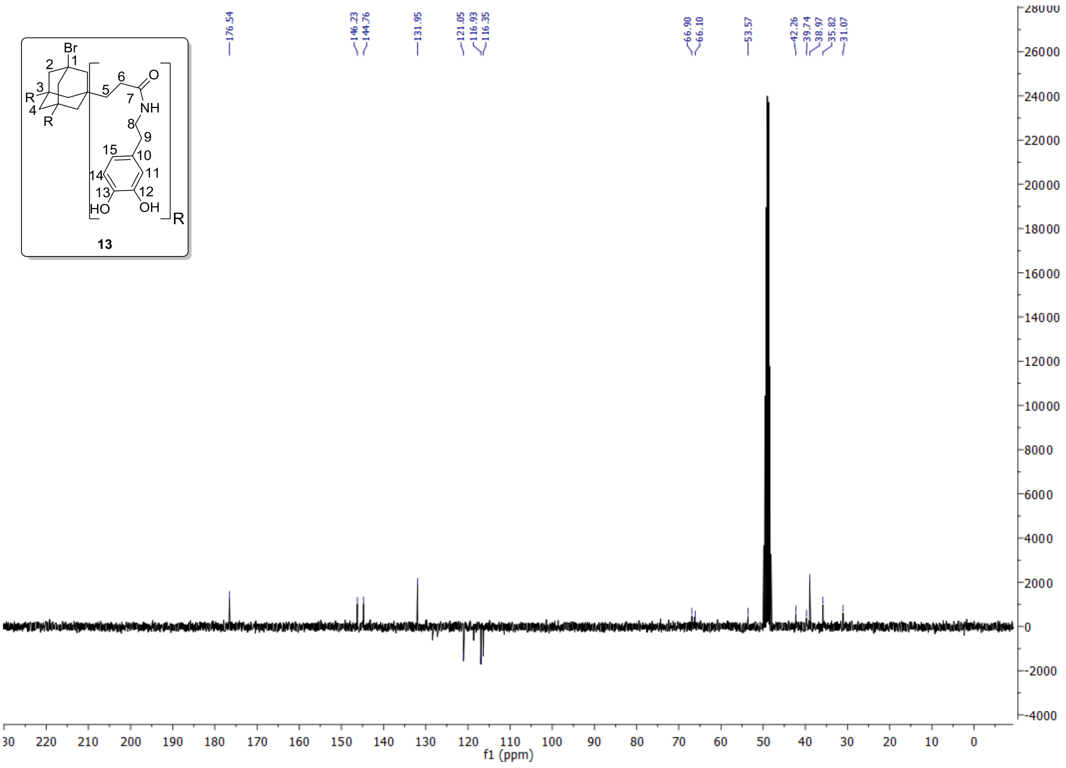

Supplementary data and general notices of the immobilization experiments

Immobilization of monomeric PEG-catecholate: 16 mg zinc oxide nanoparticles (NPs) (calculated surface area according to TEM is $2.85 \cdot 10^{18}$ nm^2) and 500 mg 1-(*N*-PEG)-dopamine (0.098 mmol) which corresponds to a five-fold excess of molecules (when taking into account that 4 molecules cover 1 nm^2) were dissolved in 0.3 mL methanol, 0.3 mL water and 6.6 mL MOPS buffer (pH 10), stirred for 12 hours at 40 °C with sonification, separated by centrifugation and washed one time with methanol (5 mL), water (5 mL) and freeze-dried for 72 h. The data were obtained at different steps of centrifugation and washing according to the notification in the appropriate plot.

Immobilization of tripodal PEG-catecholate: 50 mg zinc oxide nanoparticles (NPs) (calculated surface area according to TEM is $8.91 \cdot 10^{18}$ nm^2) and 431 mg PEG-Triscatechol (74.1 µmol) which corresponds to a five-fold excess of molecules (when taking into account that 1 molecule covers 1 nm^2) were dissolved in 1 mL methanol, 1 mL water and 20 mL MOPS buffer (pH 10), stirred for 12 hours at 40 °C with sonification, separated by centrifugation and washed one time with methanol (5 mL), water (5 mL) and freeze-dried for 72 h. The data were obtained at different steps of centrifugation and washing according to the notification in the appropriate plot.

Immobilization of bromotriscatecholate: 50 mg zinc oxide nanoparticles (NPs) (calculated surface area according to TEM is $8.91 \cdot 10^{18}$ nm^2) and 50 mg of bromotriscatecholate (59.7 µmol) which corresponds to a four-fold excess of molecules (when taking into account that 1 molecule covers 1 nm^2) were dissolved in 1 mL methanol, 1 mL water and 20 mL MOPS buffer (pH 10), stirred for 12 hours at

40 °C with sonification, separated by centrifugation and washed three times with methanol (5 mL), water (5 mL) and freeze-dried for 72 h.

Preparation of MOPS-buffer (pH 10):

2.09 g 3-morpholinopropanesulfonic acid sodium salt (MOPS), 3.50 g NaCl and 10.46 g K_2SO_4 were dissolved in 100 mL deionized water.

SEM-EDX data of the pure ZnO particles:

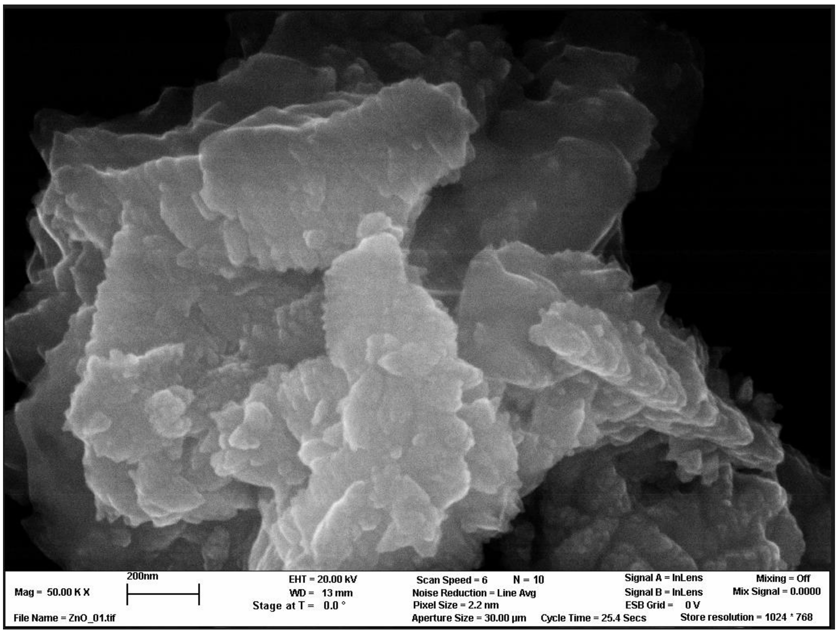

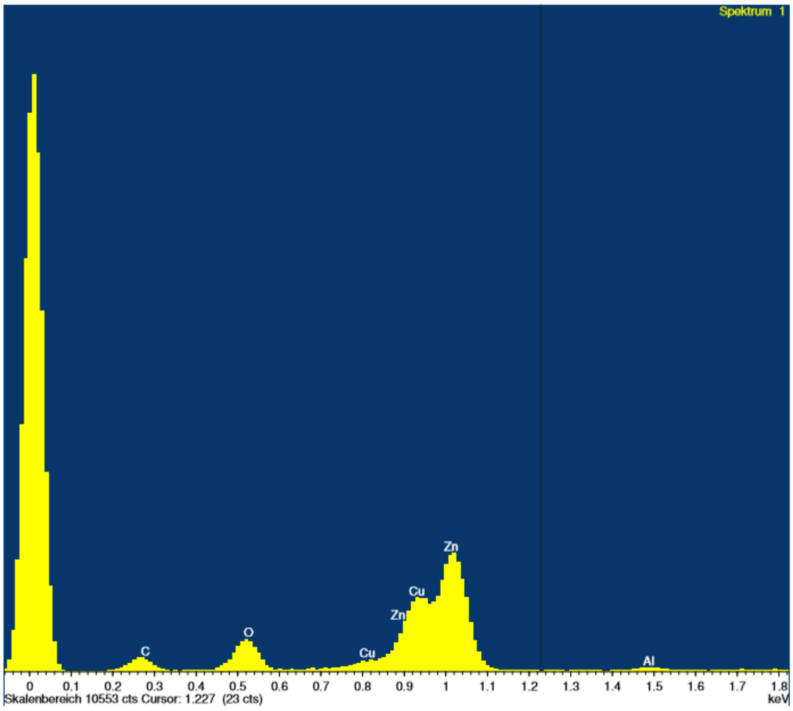

SEM and EDX Analysis: For SEM and energy-dispersive X-ray analysis (EDX), the dried particles were transferred to a carbon conductive tab and fixed on an Al specimen stub. The samples were analyzed using a Zeiss Leo 1550 Gemini microscope with field emission gun (FEG) and an Oxford EDX-System.

Data for pure molecules:

For 3-morpholinopropanesulfonate sodium:

XRD pattern:

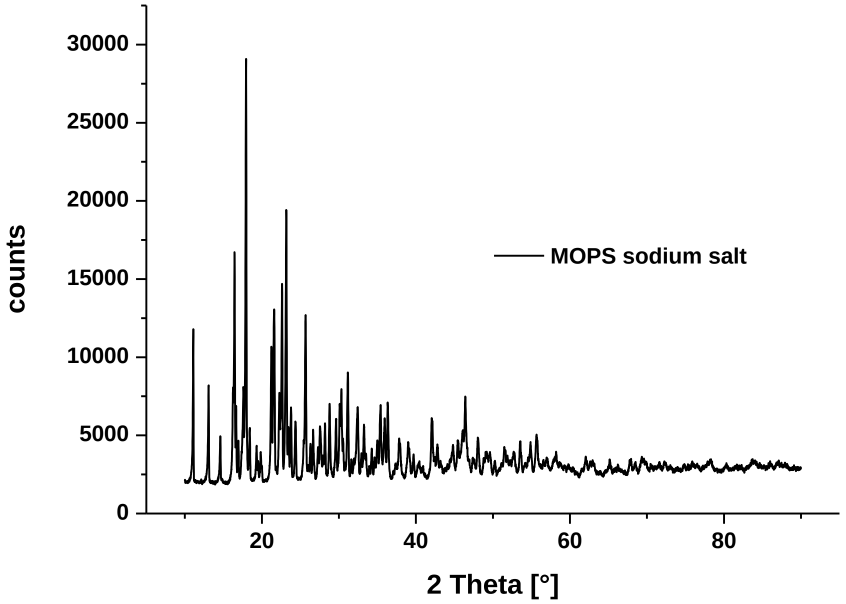

IR spectra:

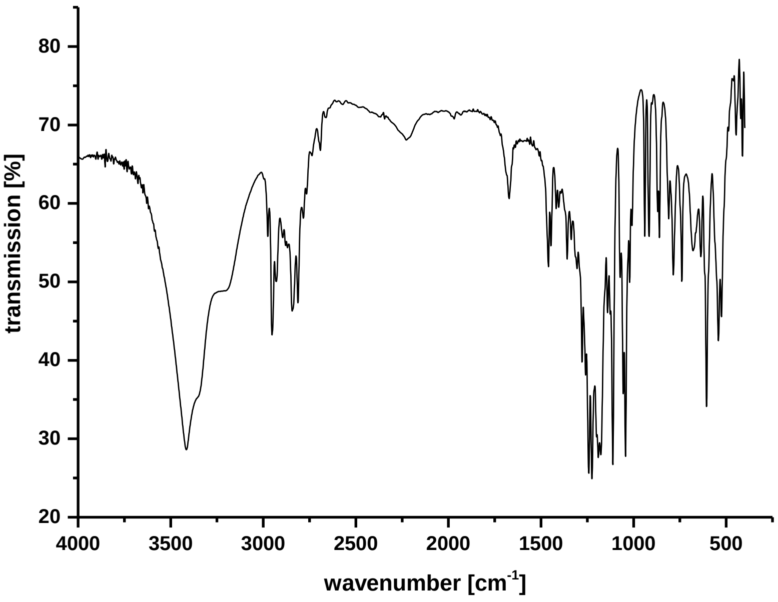

TGA:

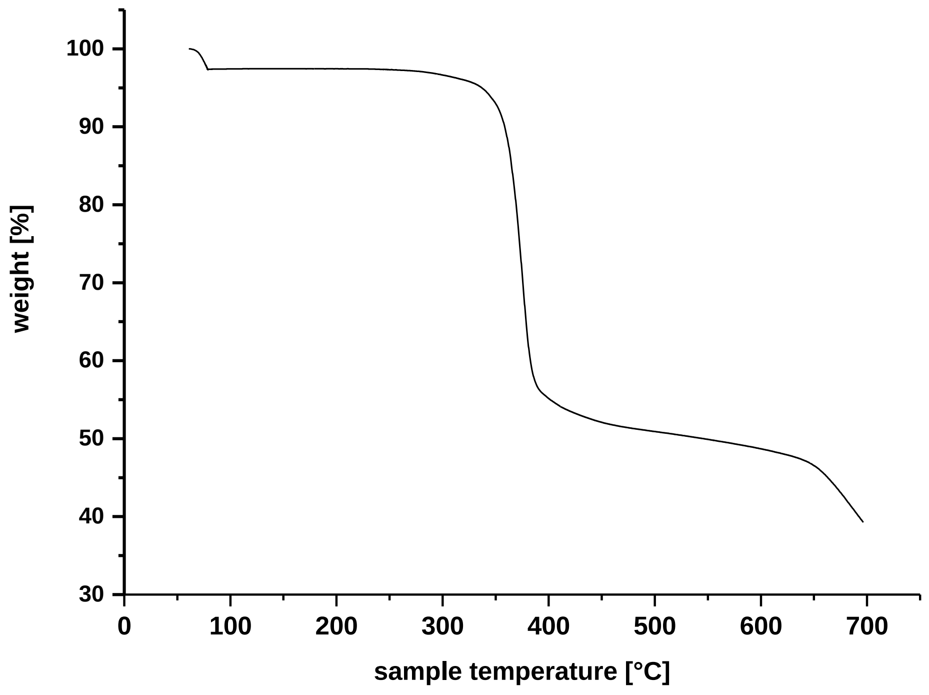

TGA curves of the pure PEG-triscatecholate:

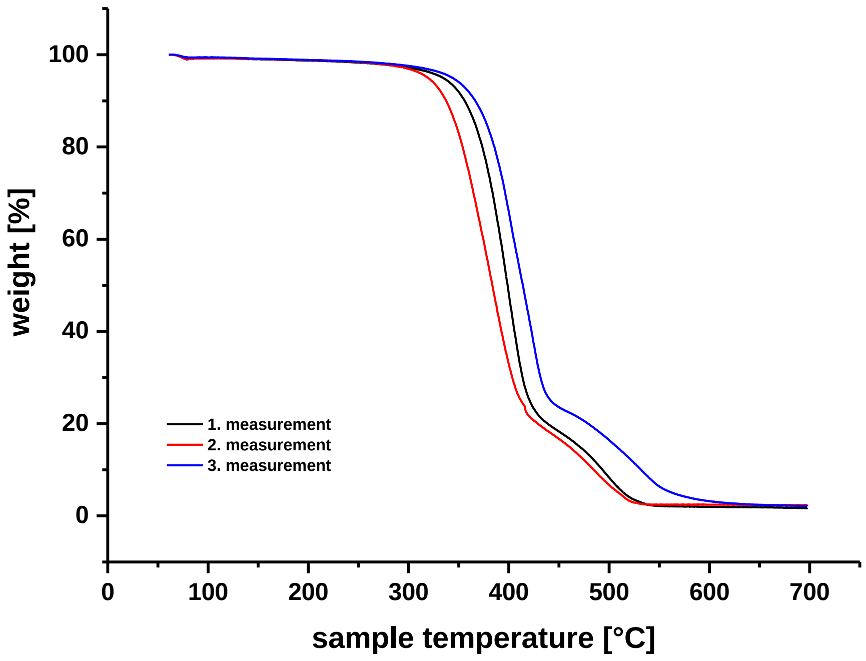

TGA curves of the pure PEG-dopamine:

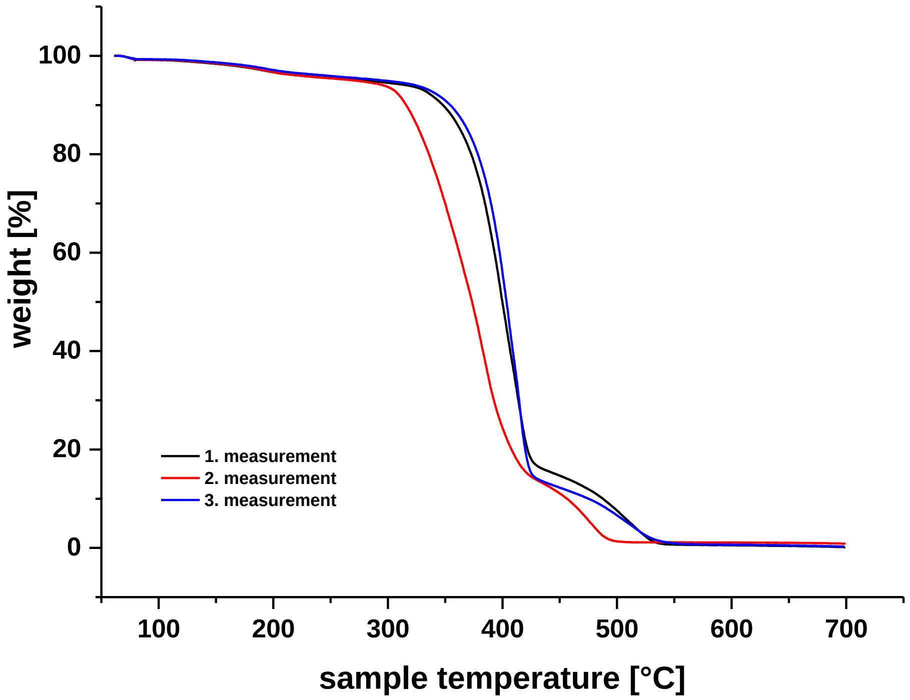

TGA curves of the pure bromotriscatecholate:

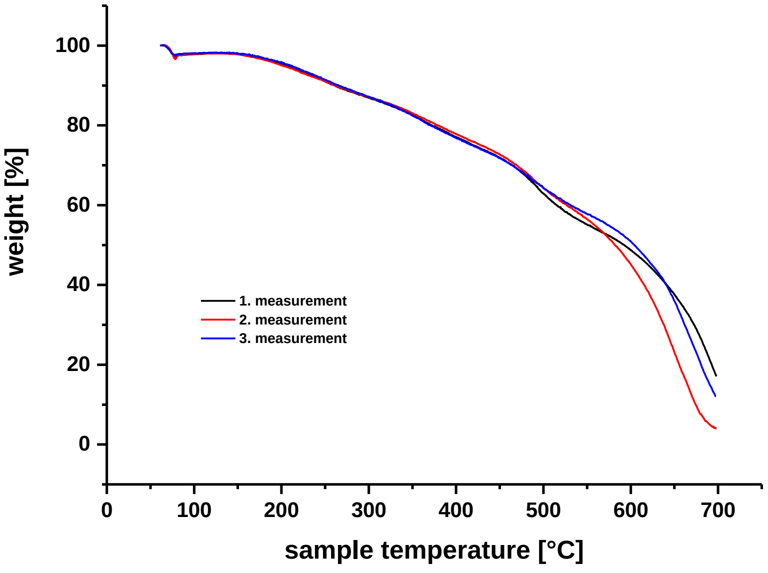

Further analytical data of the immobilized samples:

Pure particles in MOPS buffer without any catecholate:

XRD pattern:

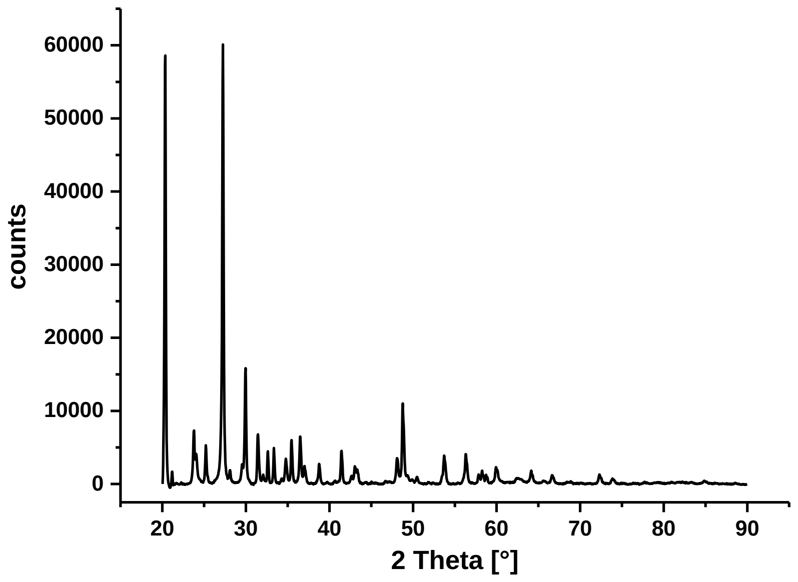

TGA

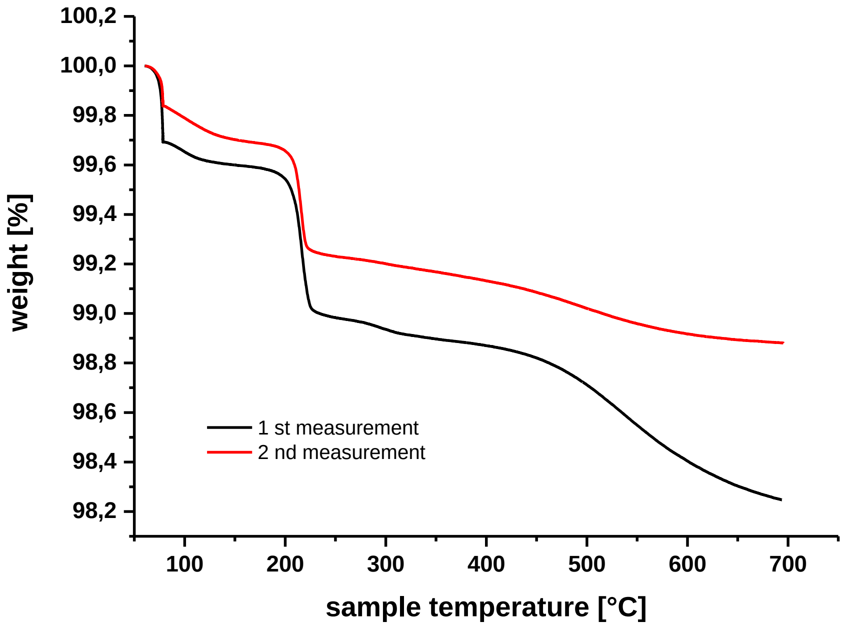

HRTEM and EDX:

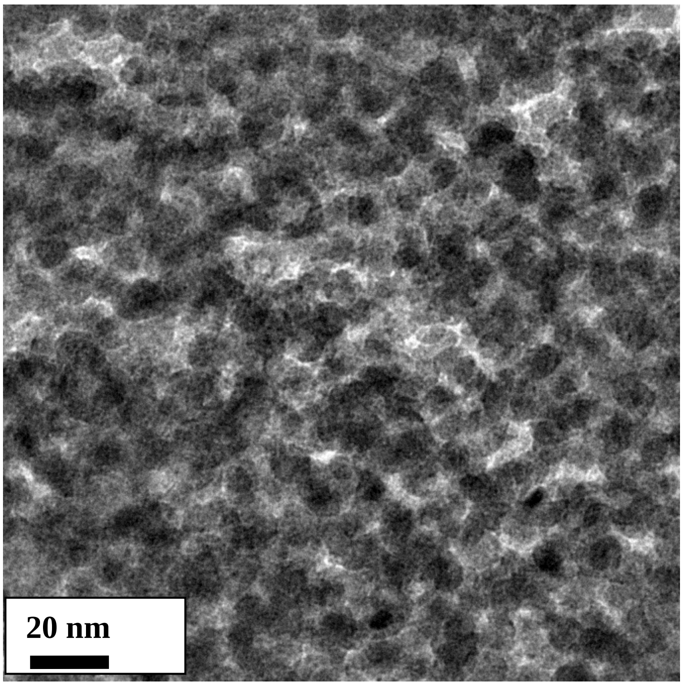

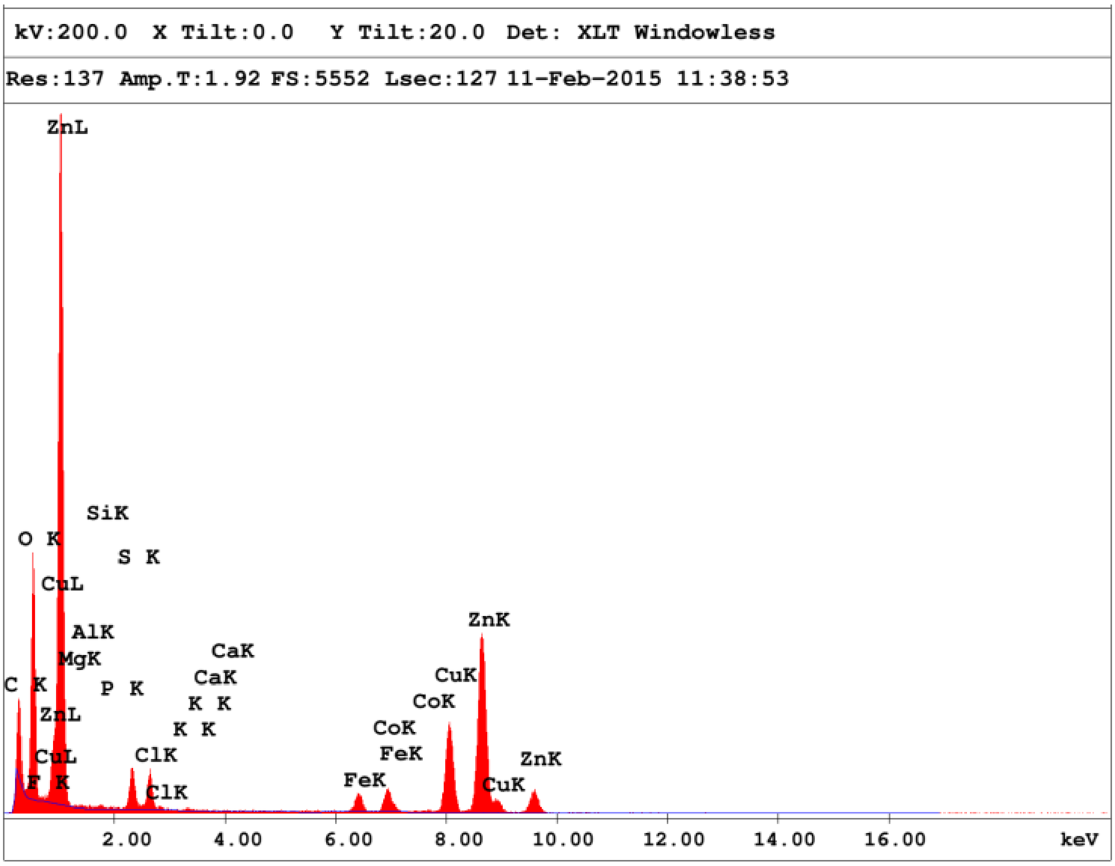

Bromotriscatecholate 13 immobilized on ZnO in MOPS buffer:

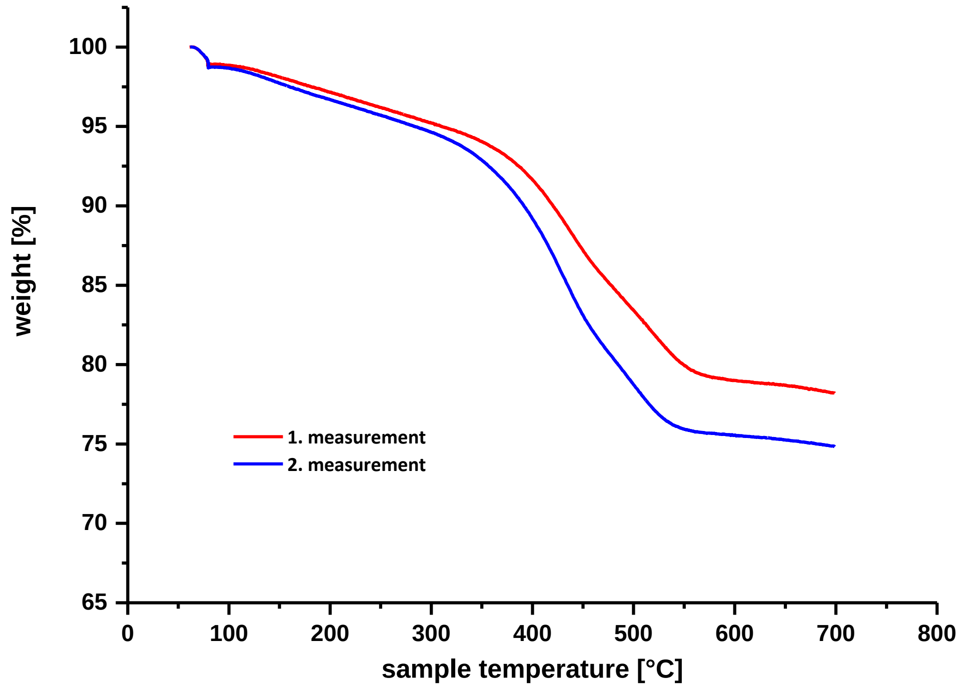

IR spectra

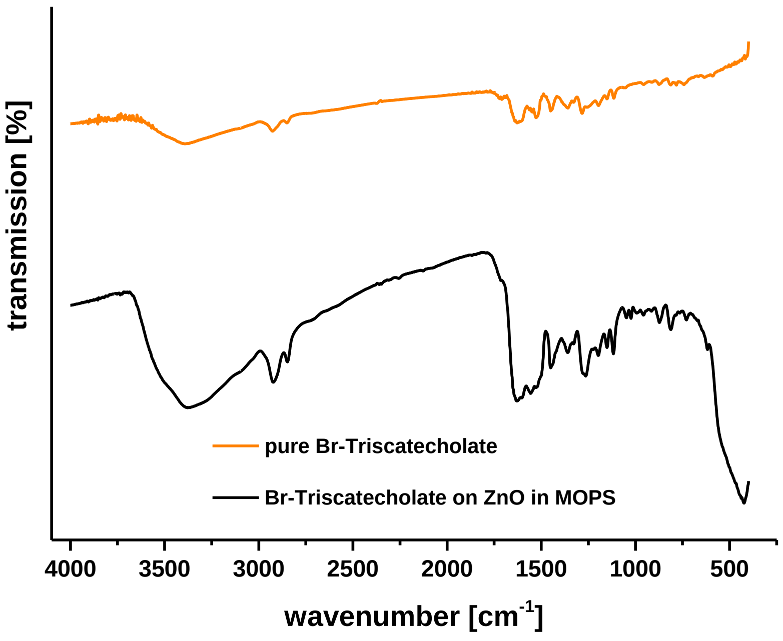

XRD pattern

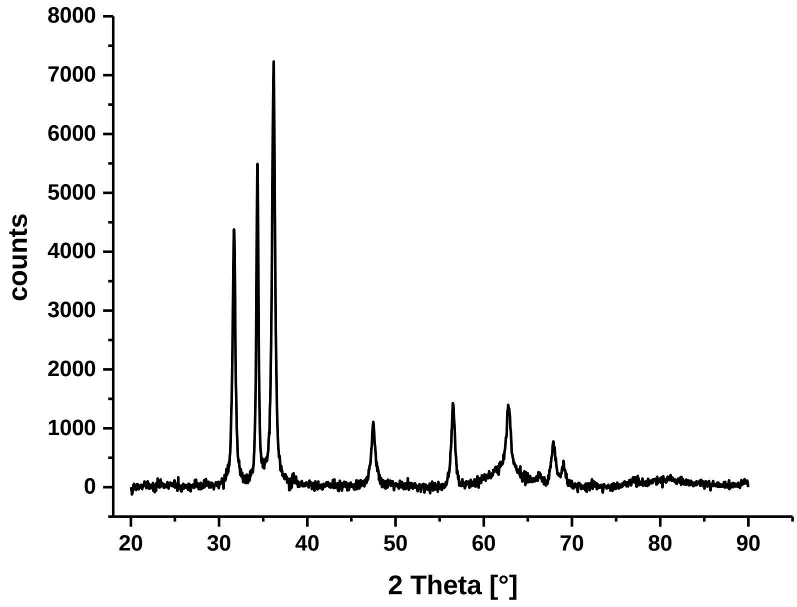

EDX data

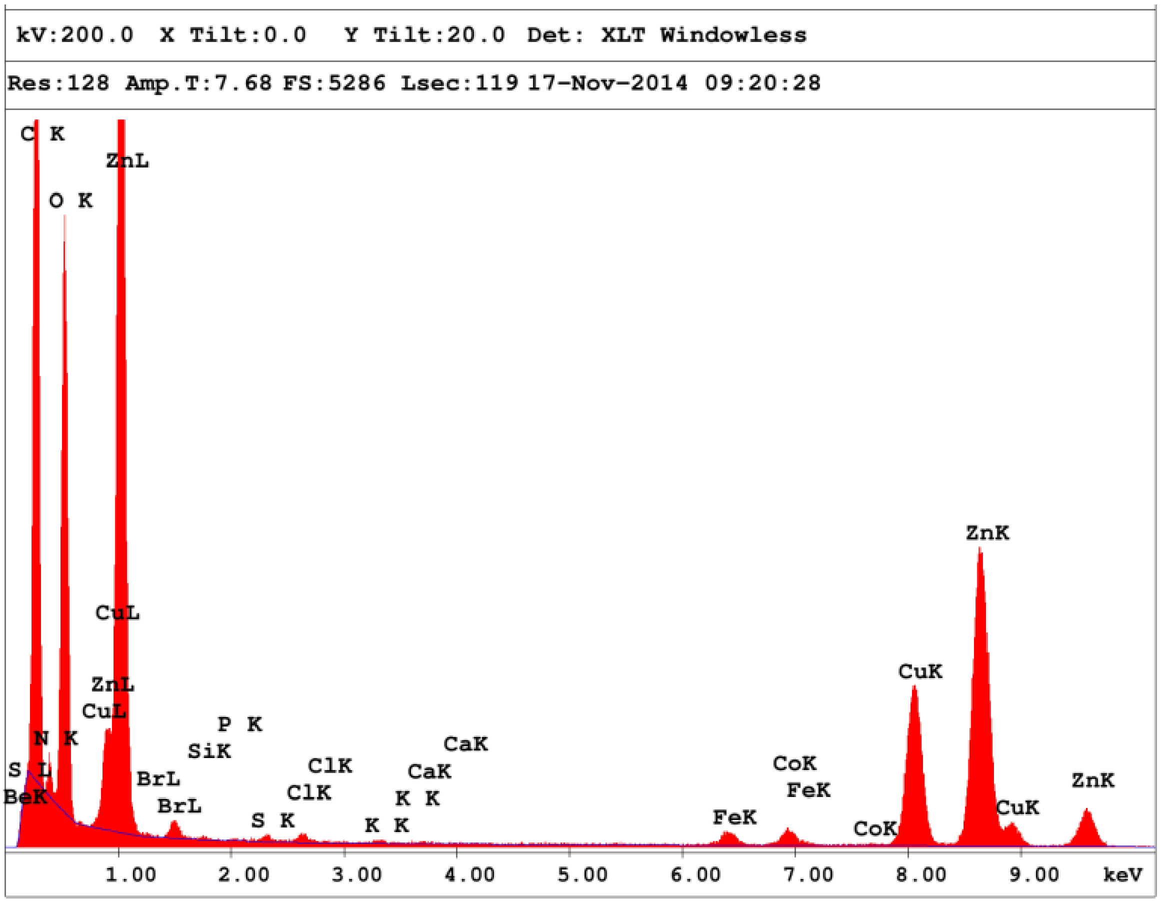

Washing experiments

PEG-dopamine 14 immobilized on ZnO

TGA of the sample centrifuged directly after immobilization:

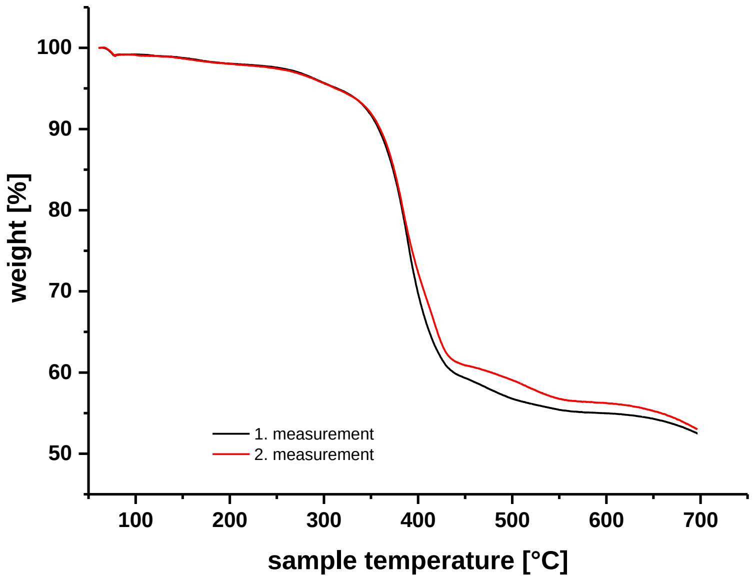

TGA of the sample after washing with water and methanol:

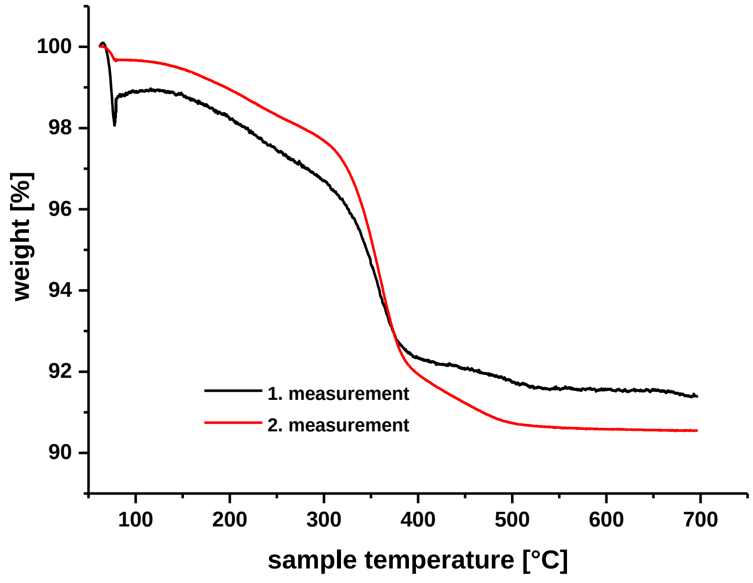

EDX of the sample after washing with water and methanol:

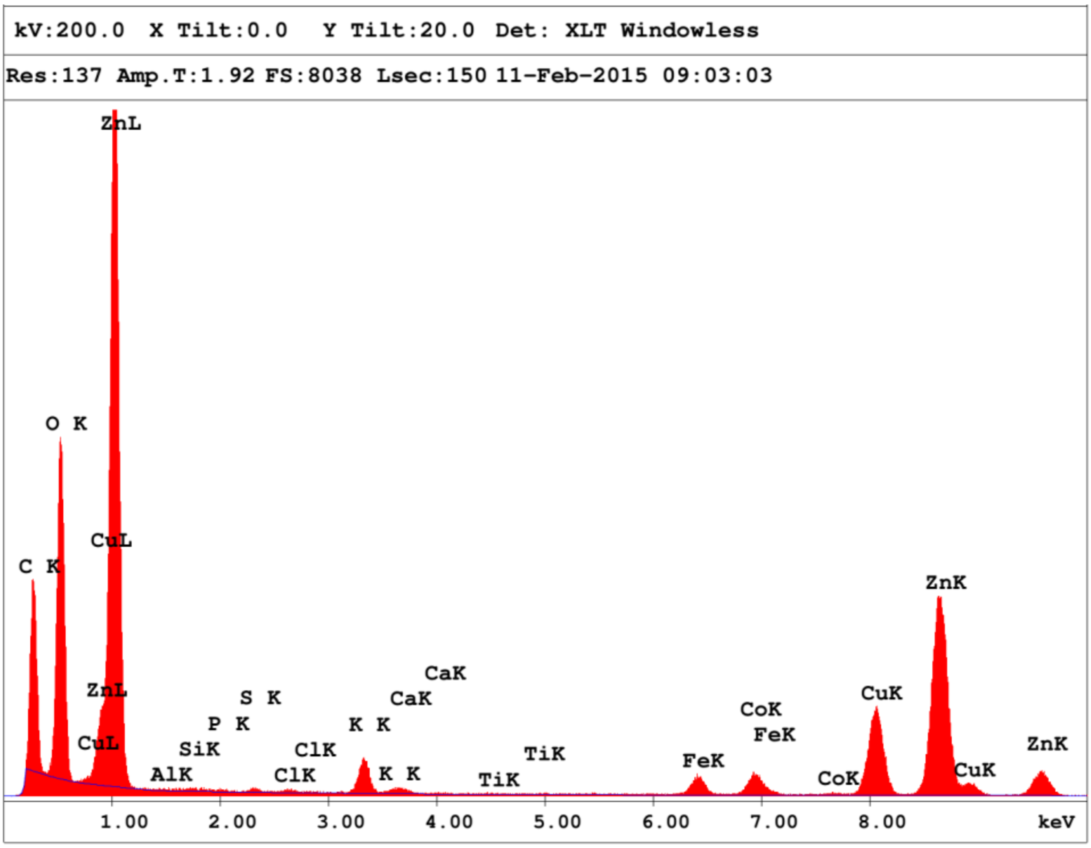

XRD pattern of the sample after washing with water and methanol:

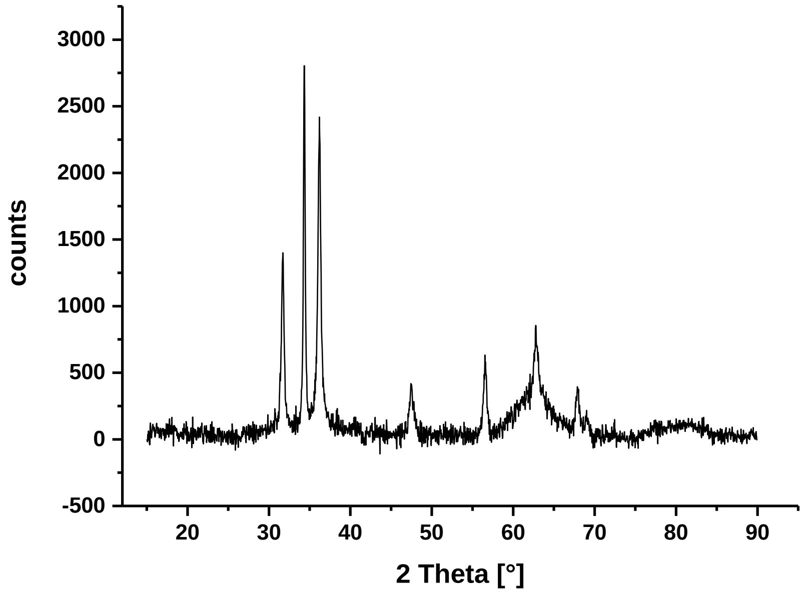

PEG-triscatecholate 3 immobilized on ZnO:

TGA of the sample centrifuged directly after immobilization:

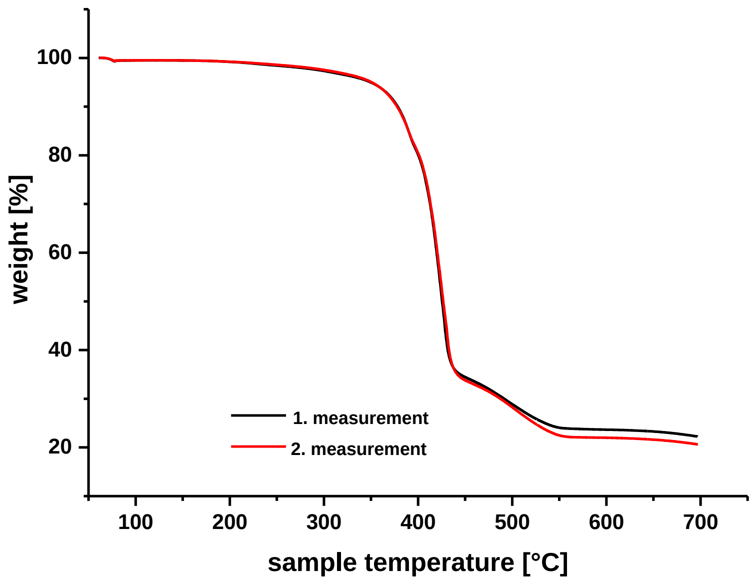

TGA of the sample after washing with water and methanol:

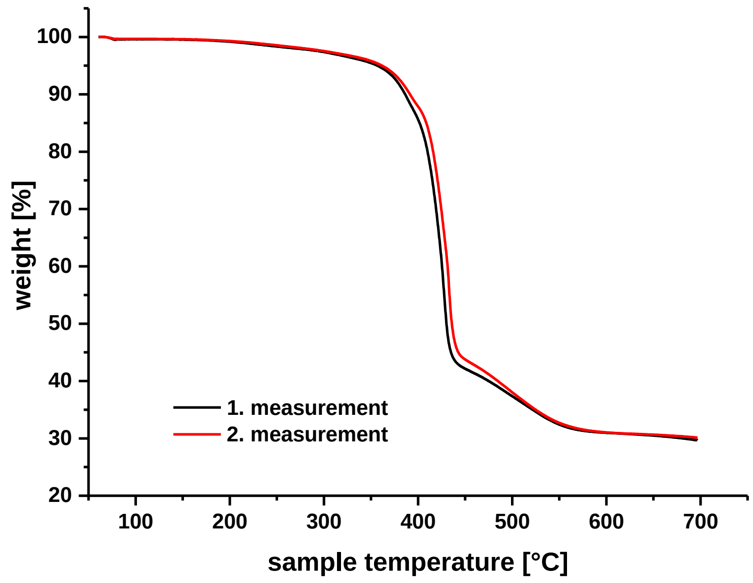

EDX of the sample after washing with water and methanol:

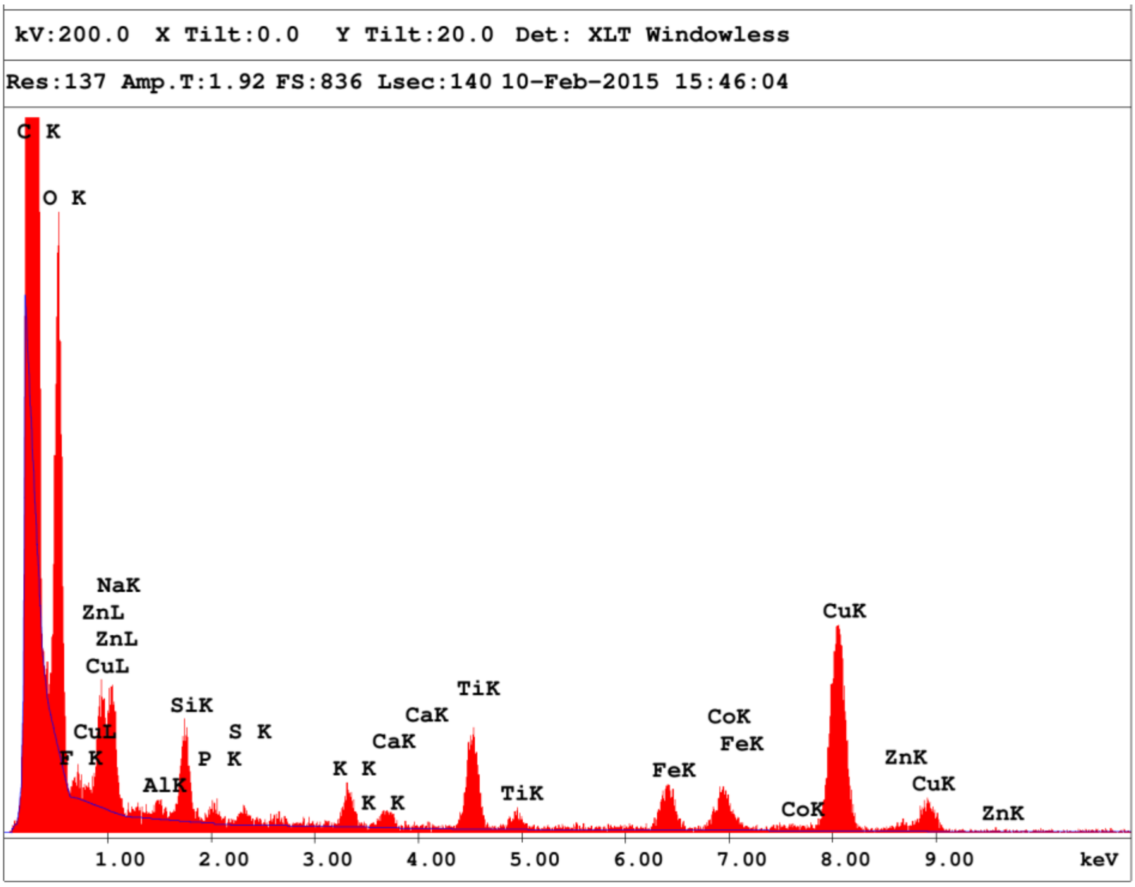

XRD pattern of the sample after washing with water and methanol:

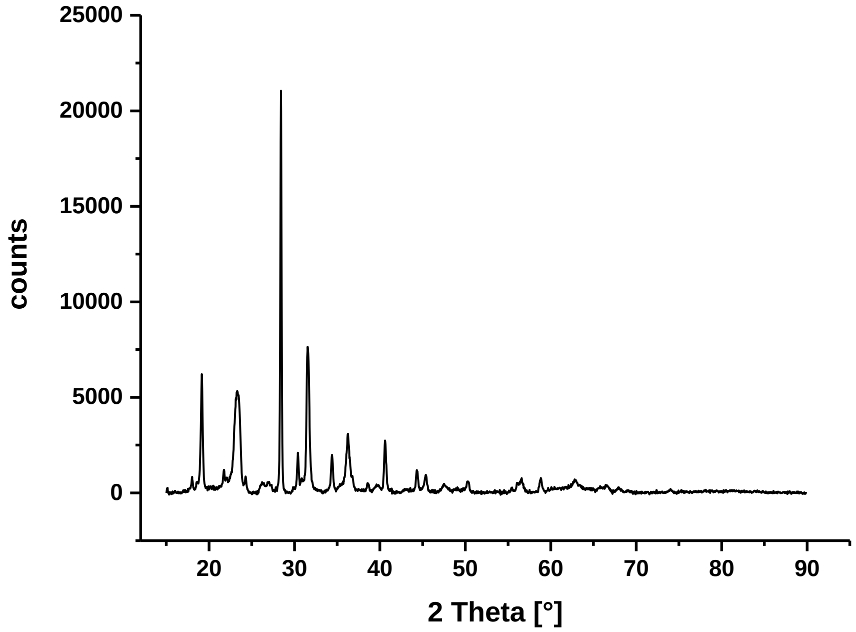

The XRD pattern shown above displays next to the signals of ZnO also signals of PEG.

IR spectra after washing with MeOH and water three times:

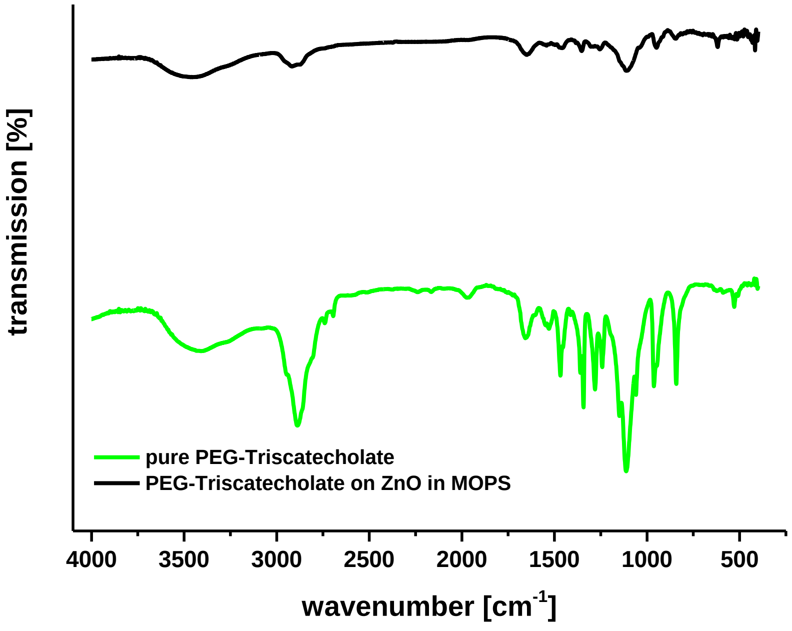

References:

1. Fleck, C.; Franzmann, E.; Claes, D.; Rickert, A.; Maison, W. *Synthesis* **2013**, 1452-1461.
2. Liu, Z.; Hu, B.-H.; Messersmith, P. B. *Tetrahedron Lett.* **2010**, *51*, 2403-2405.
3. Claes, D.; Holzapfel, M.; Clausen, N.; Maison, W. *Eur. J. Org. Chem.* **2013**, 6361-6371.
4. Pannier, N.; Maison, W. *Eur. J. Org. Chem.* **2008**, 1278-1284.
